# Psoriasis Triggers and Disease Activity: Analysis of Survey Data from the PSODEEP1 Study

**DOI:** 10.2340/actadv.v106.adv-2025-0167

**Published:** 2026-06-01

**Authors:** Albert Duvetorp, Juul van den Reek, Juni Wikström, Magnus Blom Petersen, Wenning Zheng, Josefin Lysell, Lone Skov, Fernando Valenzuela, Beatrice Dyring-Andersen, Liv Eidsmo

**Affiliations:** 1 Department of Immunology and Microbiology, LEO Foundation Skin Immunology Research Center, University of Copenhagen, Copenhagen, Denmark; 2 Department of Clinical Sciences Malmö, Lund University, Lund, Sweden; 3 Department of Dermatology and Venereology, Skåne University Hospital, Region Skåne, Sweden; 4 Department of Dermatology, Radboud University Medical Centre (RadboudUMC), Nijmegen, The Netherlands; 5 Department of Dermatology and Venereology, Sunderby Hospital, Norrbotten County Council, Luleå, Sweden; 6 The Parker Institute, Bispebjerg and Frederiksberg Hospital, Copenhagen, Denmark; 7 Department of Medicine Solna, Division of Dermatology and Venereology, Karolinska Institutet, and Karolinska University Hospital, Stockholm, Sweden; 8 Department of Dermatology and Allergy, Copenhagen University Hospital – Herlev and Gentofte, Copenhagen, Denmark; 9 Department of Clinical Medicine, Copenhagen University, Copenhagen, Denmark; 10 Department of Dermatology, Clinical Hospital of the University of Chile, Santiago, Chile; 11 Department of Dermatology, Zealand University Hospital, Roskilde, Denmark; 12 A*STAR Skin Research Labs (A*SRL), Agency for Science, Technology and Research Institute of Singapore (SRIS), Singapore, Republic of Singapore

**Keywords:** precipitating factors, psoriasis, recurrence, symptom flare up

## Abstract

Numerous patients with psoriasis will associate disease triggers to changes in disease activity over time. Modern treatments induce remission, but relapse is common if the treatment is stopped. Exploring patients’ first-hand experiences is a key starting point to identify triggers that may influence disease relapse. A cross-sectional digital questionnaire was distributed to 2716 individuals with psoriasis in Chile, Denmark, Sweden and the Netherlands. 71% of participants reported psoriatic disease triggers. Triggers were classified into 24 subgroups dominated by stress (55.3%), infections (17.7%) and alcohol (10.8%). The majority (65%) of the participants experienced seasonal variation in disease activity, and approximately half reported fluctuating disease activity with periods of flare-ups. The self-reported trigger prevalence varied by gender, disease manifestation (skin psoriasis and/or psoriatic arthritis) and country of residence. The female participants more often reported stress and infections as triggers. Alcohol consumption is more often reported as a trigger in males. Chileans more often reported stress and less frequently reported infections as triggers. Insights into patient-reported disease variations and triggers can be used to aid patient-physician conversations and patient phenotyping during routine clinical practice. Trigger assessment and management could potentially contribute to patient counselling and motivate lifestyle changes.Clinicaltrials.gov listing: NCT24435875.

**SIGNIFICANCE**

To better understand dynamics of psoriasis disease activity, there is a need to map trigger factors. This study provides data on how common self-reported triggers are (classified into 24 subgroups) and how the disease can fluctuate. To individualize the clinical care of patients with psoriasis, there is a need to understand common patterns. For example, patients with a seasonal influence on disease activity may require more intensive treatment during the season of disease worsening. Study results can be used to facilitate dialogue with patients in daily medical practice and to design future studies on triggers and underlying disease biology.

Although psoriasis is a chronic condition, patients often experience fluctuating disease activity and variable disease presentations over time. In the 10 year follow-up of the Stockholm Psoriasis Cohort, 48% of individuals with guttate psoriasis and 20% of individuals with plaque psoriasis exhibited minimal disease activity at follow-up (1). While the precise reason for the favourable long-term prognosis of patients with guttate psoriasis in comparison to plaque psoriasis is unclear, an intermittent disease trigger, streptococcal infection, is strongly associated with guttate onset and relapse (2). Previous studies investigating psoriasis triggers have reported a range of triggers across different populations. In the German ActiPso study, activity types for psoriatic disease were determined, resulting in the following classification: “stable” (type 1), “unstable” (type 2), “winter type” (type 3) or “summer type” (type 4). Within these types, the proportion of patients experiencing a worsening of their psoriasis due to triggers differed. Type 3 followed by type 2 reported the highest trigger prevalence (73.8% and 67.8% respectively). Altogether, 54.7% of participants reported stress, 19.5% reported infections as a trigger, and 36.57% classified their psoriasis as seasonally influenced (winter/summer) (3). In contrast, in a large Chinese study of over 12,000 patients with psoriasis, 34.5% of the participants reported stress, 27.4% reported infection, and 60.2% reported season as disease triggers (4). Additional triggers reported in the literature include obesity/weight gain (5), alcohol (4), air pollution (6), the Koebner phenomenon, climate and drugs (7).

Trigger studies commonly have a list of specific triggers from which participants can choose, which introduces confirmation bias. Variation in study design also diminishes the validity of comparisons between study populations. In the current study, we used an exploratory open-question approach and performed the study across four different countries on two continents to allow for better comparison. This study is clinically highly relevant as patients often inquire about the factors that they suspect have contributed to their disease onset or influenced their disease flare-ups.

## MATERIALS AND METHODS

### Data collection

**Fig. 1.**
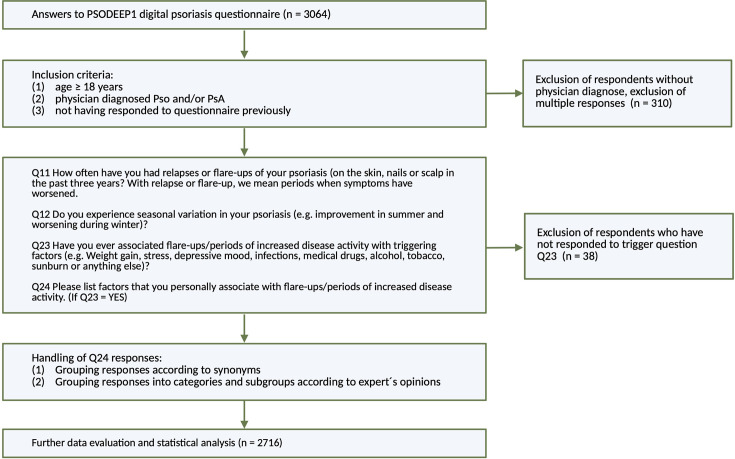
Flowchart of methods.

The PSODEEP1 digital questionnaire was designed to investigate self-reported disease triggers, disease fluctuation, occurrence of the Koebner phenomenon and symptoms of psoriatic arthritis in patients with psoriasis. PSODEEP1 was administered to patients in Chile, Denmark, the Netherlands and Sweden, between October 2022 and February 2024. Questions (Appendix S1) were translated into the local dominant languages (Spanish, Danish, Dutch, Swedish) and adapted to country-specific cultural interpretations by Fernando Valenzuela (FV), Lone Skov, Juul van den Reek (JVDR) and Albert Duvetorp (AD). The questionnaire was distributed to patients in dermatology and rheumatology outpatient clinics and through patient organizations active in the four countries (*Fundación Padece*, *Psoriasisforeningen*, *Psoriasispatiënten Nederland*, *Psoriasisförbundet* and *Ung med Psoriasis*). Patient organizations posted information about the study on social media, printed media, emails and homepages. Study questions were answered by scanning QR codes, most often with the study participants’ own smartphones. Data were collected and managed using REDCap electronic data capture tools (8) hosted at Lund University for EU data and at the Universidad de Chile for data from Chile. The inclusion criteria were as follows: minimum age of 18 years, informed consent and physician-diagnosed skin psoriasis (Pso) and/or psoriatic arthritis (PsA). Fig. 1 provides a schematic overview of the study. Participants who reported having experienced flare-ups secondary to disease triggers were asked to list their trigger experiences in an open text field. The answers were translated into English and categorized. Open-text descriptions of triggers needed to be interpreted for categorization. To minimize introduction of interpretation bias, a panel approach to interpretation was adopted for unclear responses. AD, JVDR, Juni Wikström and FV categorized triggers after discussion, considering both language and contextual factors. In the case of the panel not reaching full consensus, the specific trigger response was sorted to an “uncategorised” category.

### Data inclusion and exclusion

Duplicate responses were identified by the submission of identical answers at the same time/date, or responses sharing the same contact email or mobile telephone number. Forty-one participants indicated that they had experienced disease triggers but did not specify any in the follow-up questions and were classified as having missing data. Fifty-two responses were not precise enough to classify into specific subgroups and were categorized as “uncategorized”.

### Statistics analysis and visualization

**Fig. 2.**
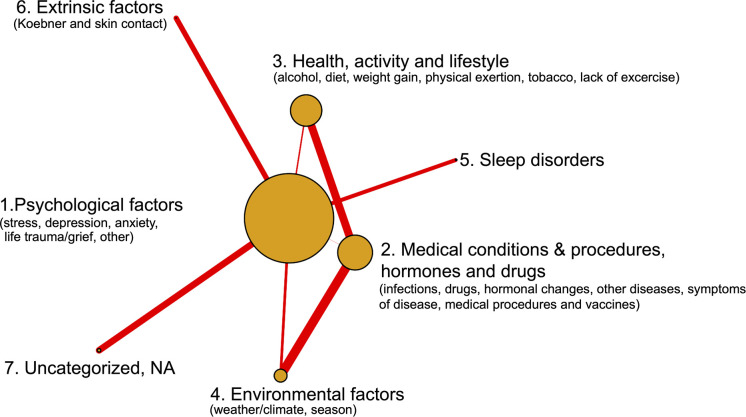
Network graph of self-reported psoriasis trigger categories. Co-occurrence is represented by edge thickness. Node size represents trigger prevalence.

Continuous variables are presented as means with 95% confidence intervals. Categorical variables are presented as percentages with numbers where appropriate. χ^2^ tests were employed to compare categorical variables between groups, with *p*-values<0.05 considered significant. Exploratory logistic regression models were used to analyze the association between self-reported disease activity Body Surface Area (BSA), gender, age, PsA presence, country of origin and the three major triggers (stress, infections and alcohol). The tested assumptions for the models were multicollinearity, outliers and whether continuous variables were related to log odds (data was log-transformed if necessary). BSA is a categorical outcome, but models are better interpretable when it is added as a continuous variable. Therefore, for each model, we tested two scenarios: BSA as a continuous variable and as a categorical variable; in the results, it is presented as continuous when both scenarios were comparable, but as categorical if this was not the case. Raw data were pooled and processed using SPSS Statistics (version 29.0.2.0). Fig. 1 was created on Biorender.com. Fig. 2 was elaborated in RStudios (Version 2023–09.1+494) using the igraph package, and all other figures were produced using GraphPad Prism (Version 10.4.0).

## RESULTS

### Baseline characteristics and demographics of participants

A total of 2,716 participants completed the PSODEEP1 questionnaire and provided analyzable responses regarding triggers and disease fluctuation. The highest number of responses were obtained from Sweden (*n*=1,714), followed by the Netherlands (*n*=421), Denmark (*n*=304) and Chile (*n*=277). Plaque psoriasis was the predominant type of psoriasis, and most participants were female (66.5%). The country-specific baseline and clinical characteristics of the patients are summarized in Table I. Many participants reported that their dominant skin psoriasis type was guttate (39%). 41% of participants reporting guttate psoriasis also reported taking systemic therapy, which suggests that individuals with small plaques may have classified themselves as having guttate-type psoriasis.

**Table I.** Characteristics of study participants

Country	Chile		Denmark		The Netherlands		Sweden		Total	
Proportion of total % (*n*)	10.2% (277)		11.2% (304)		15.5% (421)		63.1% (1,714)		100% (2,716)	
Female % (*n*)	49.8% (138)		75.3% (229)		82.4% (347)		63.8% (1,093)		66.5% (1,807)	
Age, mean (SD)	43.0 (±13.1)		51.1 (±15.1)		50.8 (±13.3)		56.8 (±15.6)		53.8 (±15.6)	
PsA % (*n*)	25.6% (71)		47.0% (143)		34.2% (144)		42.2% (723)		39.8% (2,716)	
Dominant type of skin psoriasis % (*n*)*	Plaque guttate pustular n.s.	51.3% (142) 32.9% (91) 5.4% (15) 10.5% (29)	Plaque guttate pustular n.s.	32.6% (99) 34.9% (106) 10.5% (32) 22.0% (67)	Plaque guttate pustular n.s.	45.8% (193) 36.1% (152) 2.4% (10) 15.7% (66)	Plaque guttate pustular n.s.	36.9% (633) 41.4% (709) 3.4% (58) 18.3% (314)	Plaque guttate pustular n.s.	39.3% (1,067) 39.0% (1,058) 4.2% (115) 17.5% (476)
Treatment % (*n*)**	Topicals Phototherapy Systemics (orals, injectables)	82.7% (229) 9.4% (26) 47.3% (131)	Topicals Phototherapy Systemics (orals, injectables)	79.3% (241) 9.9% (30) 48% (146)	Topicals Phototherapy Systemics (orals, injectables)	71.7% (302) 15.2% (64) 42.5% (179)	Topicals Phototherapy Systemics (orals, injectables)	84.1% (1,442) 14.8% (254) 48.6% (833)	Topicals Phototherapy Systemics (orals, injectables)	81.5% (2,214) 13.8% (374) 47.5% (1,289)

* As defined by participants. ** Participants can have multiple treatments.

n.s.: not specified – do not know; PsA: psoriatic arthritis; SD: standard deviation.

### Dynamics of disease activity and seasonal variation in psoriasis

A total of 2,455 individuals responded to the questions regarding disease flare-up frequencies over the past 3 years (defined as “periods when symptoms have worsened”). Among these responders, 38% reported constant symptoms with no remission periods, whereas 10% reported no flare-ups or relapses, which was defined as sustained remission. The remaining 49% experienced fluctuating disease activity, with flare-ups occurring at frequencies ranging from weekly to monthly intervals, to as infrequently as once every 3 years (Fig. S1). An average of 65% of participants from all 4 countries reported seasonal variation in disease activity, with similar frequencies (Chile 61.4%, Denmark 60.5%, the Netherlands 62.1% and Sweden 67.2%) (Fig. S2).

### Trigger factors for psoriasis flare-ups

Among all responders, 71% reported experiencing triggers of disease activity. Triggering factors are defined as those associated with “flare-ups/periods of increased disease activity”. Most participants reported 1–3 triggers (Fig. S3). Reported triggers were classified into 7 overarching categories (categories 1–7) and 24 subgroups (subgroups A–X in Table II). Panel consensus (inter-rater agreement) was 97.3%. To estimate the reproducibility of panel classification, a psoriasis expert MD uninvolved in the project was asked to reclassify 220 responses, leading to a reproducibility of 96%. The first 50 individual responses in their original language, along with their translation and classification, can be found in Appendix S2. The interrelationships among the seven categories are illustrated in the network diagram (Fig. 2).

**Table II.** Self-reported psoriasis disease triggers. Responses are divided into subgroups (A–X) with overarching categories (1–7)

Categories	Number (*n*)	Gender (%)		All (%)	*Subgroups*	Number (*n*)	Gender (%)		All (%)
		Female	Male				Female	Male	
1. Psychological factors	1,557	60.9	50.3	57.3	A. StressB. DepressionC. AnxietyD. Other psychologicalE. Life trauma/grief	150221810967 66	59.28.64.42.7 2.8	47.66.93.32.11.8	55.38.04.02.52.4
2. Medical conditions, medical procedures, hormones and drugs	667	29.3	14.9	24.5	F. Infections G. Drugs H. Hormonal changes I. Medical conditions/other disease unspecified J. Symptoms of disease/malaise K. Medical procedures L. Vaccinations	482776968391816	21.13.23.83.21.70.70.7	10.92.10.11.11.00.70.4	17.72.82.52.51.40.70.6
3. Health, activity and lifestyle	608	21.7	23.9	22.4	M. Alcohol N. Diet O. Weight gain P. Physical exertion Q. Tobacco R. Lack of exercise	294248149624943	9.410.15.12.41.81.5	13.87.16.32.11.91.8	10.89.15.52.31.81.6
4. Environmental factors	304	12.9	7.9	11.2	S. Weather/climate T. Season	193112	8.64.3	4.13.8	7.14.1
5. Sleep disorders	118	4.5	4.1	4.4	U. Sleep disorders	118	4.5	4.1	4.3
6. Extrinsic factors	97	4.0	2.7	3.6	V. Koebner W. Skin contact	7329	2.91.2	2.20.8	2.71.1
7. Uncategorized or missing data	91	3.4	3.4	3.4	X. Uncategorised Y. Missing data (not answered)	5141	1.81.6	2.11.3	1.91.5

### Psychological factors

In line with previous studies (3, 9), psychological factors emerged as the most common psoriasis trigger (57.3%), with stress specifically mentioned by 55.3% of the study participants. While some stress responses were descriptive, such as high workload, the majority simply indicated “stress” without elaboration. Many participants reported traumatic life events, including divorce, death of relatives and grief, as triggers. These were categorized in an individual subgroup: “life trauma/grief” (subgroup E). Responses reporting “mood”, “emotions” or “emotional state” without further specification of the type of mood or responses of specific emotions such as “anger” were classified as “other psychological” (subgroup D).

### Medical conditions, medical procedures, hormones and drugs

Several female participants reported “hormonal changes” as a trigger whereas others specified menstruation cycles, birth control pills, pregnancy, childbirth, menopause, breastfeeding or puberty as influencing disease activity and these responses were labelled “hormonal changes” (subgroup H). Participants reporting “other disease”, “being sick”, “sickness”, or symptoms of sickness such as “fatigue”, or “having inflammation in the body” were categorized separately from triggers associated with tissue harming interventions such as surgeries and dental procedures as triggers (subgroups I, J and K respectively). Vaccines were reported as triggers by 16 participants, 15 of whom specifically mentioned COVID vaccines.

### Health, activity and lifestyle

Alcohol consumption was the third most reported trigger, with 10.8% of participants identifying it as a disease trigger. Regarding “diet” (subgroup N), many participants reported “poor diet”, “poor nutrition”, “bad diet”, “high sugar intake”, “fried food” while a few reported specific food items – “gluten” and “sugar” being the most common. “Weight gain” (subgroup O) was reported as a trigger by 5.5% of participants. “Physical exertion” (subgroup P) was predominantly reported as a trigger of psoriasis among individuals with PsA (88.7% of *n*=62 responders with this trigger). “Tobacco” use (subgroup Q) and “lack of exercise” (subgroup R) were reported as disease triggers by 1.8% and 1.6% of participants, respectively.

### Environmental factors

Sudden changes in weather or specific weather conditions such as “cold” or “temperatures below 0” were reported by 7.1% – “weather” (subgroup S) – while seasonal related responses (predominantly winter) were reported by 4.1% “season” (subgroup T). Reporting weather as a trigger was more commonly reported among individuals with PsA than among those with Pso alone (9.0% vs 5.9%, χ^2^ *p<0.01*).

### Sleep disorders

“Sleep disorders” were reported as a trigger by 4.3% of the participants and were generally described as “bad sleep”, “poor sleep” without specifying the cause (subgroup U).

### Extrinsic factors

Development of psoriasis secondary to skin injury - “Koebner” phenomenon responses (subgroup V, 2.7%) were generally descriptive such as “wounds”, “skin injury” and “sunburn”. When it was unclear whether skin contact led to skin injury or if the descriptions could not rule out contact allergy, the response was categorized as skin contact (1.1%) (subgroup W). The low number of individuals listing “Koebner” as a trigger could imply that the Koebner phenomenon is not widely perceived as a trigger associated with flare-ups as stated in the questionnaire question.

### Gender, psoriatic arthritis, disease activity, nationality of participants and disease triggers

**Fig. 3.**
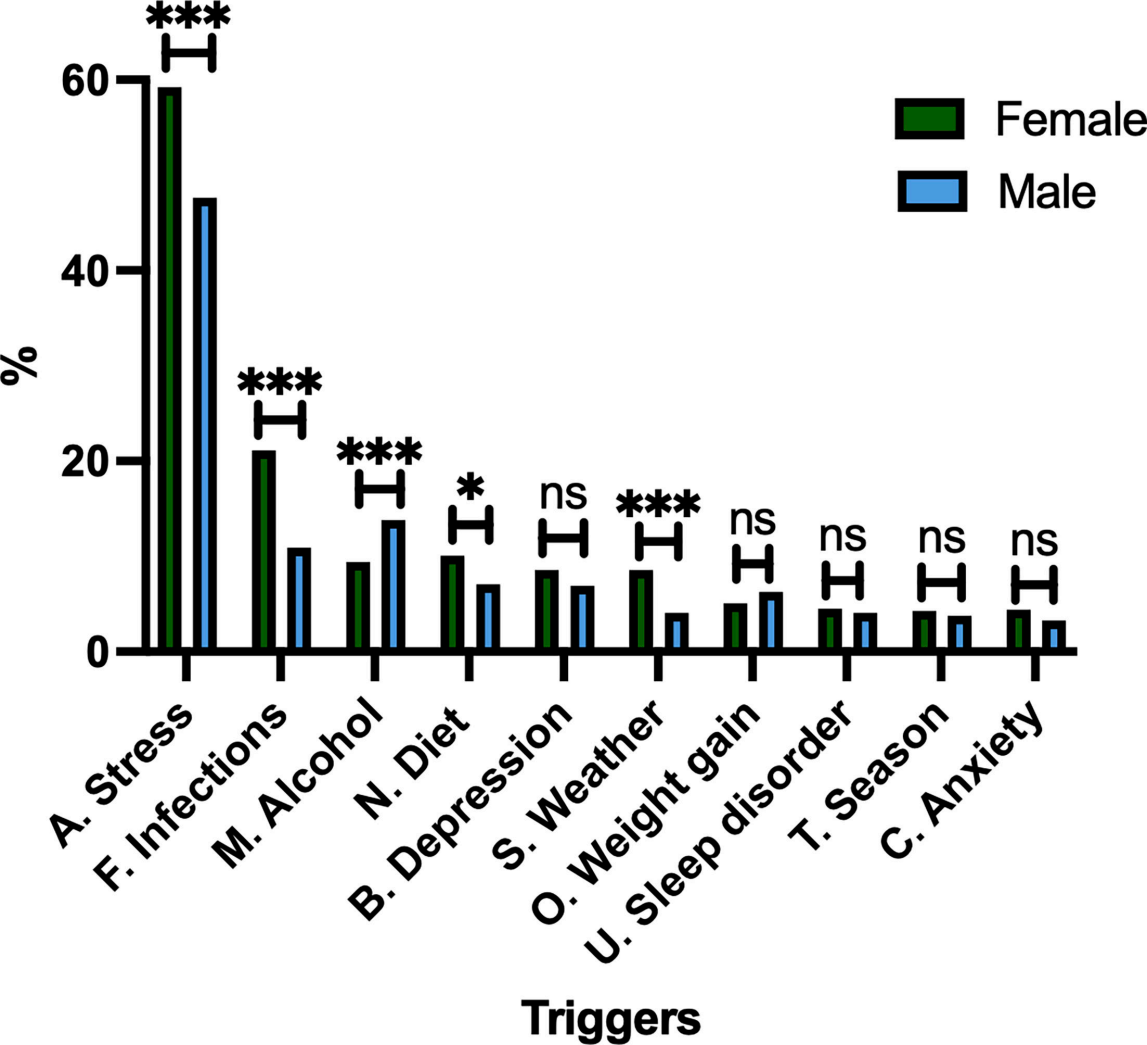
Bar chart of top 10 reported trigger categories divided by male vs female participants. χ^2^ test, *p<0.05, ***p<0.001. After Bonferroni correction (10 tests); stress, infections, alcohol and weather remain significant but diet is nonsignificant.

Next, we performed logistic regression models to analyse the three most prevalent trigger subgroups: stress (subgroup A), infections (subgroup F), and alcohol (subgroup M) in relation to country, skin disease activity (reported as BSA), PsA, gender and age (Table III). Notably, Chilean origin was significantly associated with reporting stress as a trigger, whereas these participants were less likely to report infections as a disease trigger. Being male, younger age, having PsA, a BSA of 20 palms or more and responding from Denmark (and Sweden) were associated with higher odds of reporting alcohol as a disease trigger. Male and female participants in this study reported similar BSA involvement; male participants were slightly older (mean age: 56 vs 53 years). A higher proportion of female participants reported having PsA than males (41.4% vs 36.6%), consistent with previously reported distributions (10). The self-reported triggers suggested several sex-related differences (Fig. 3). Female participants more frequently reported stress (59.2% vs 47.6%), infections (21.1% vs 10.9%), diet (10.1% vs 7.1%) and weather (8.6% vs 4.1%) as triggers than male participants. Conversely, men reported alcohol as a disease trigger more often than females (13.8% vs 9.4%). Participants with PsA were slightly older than those without PsA (mean age, 56 vs 52 years). PsA is also associated with different self-reported trigger patterns (Fig. S4). Among the top 10 trigger subgroups, participants with PsA more often reported infections (20.1% vs 16.1%), diet (11.6% vs 7.5%), weight gain (6.7% vs 4.7%), depression (10.4% vs 6.5%), and sleep disorders (5.6% vs 3.5%) among the top 10 trigger subgroups.

**Table III.** Multivariable logistic regression models of top 3 triggers: stress, infections and alcohol

Trigger: Stress				Trigger: Infections			Trigger: Alcohol			
Analysis characteristic	β coefficient	OR (95% CI)	*p*-value	β coefficient	OR (95% CI)	*p*-value	Analysis characteristic	β coefficient	OR (95% CI)	*p*-value
Age	−0.88	0.42 (0.32–0.54)	<0.001 ***	−0.01	0.99 (.99–1.00)	0.097 (ns)	Age	-0.04	0.97 (0.96–0.97)	<0.001***
Male gender	−0.48	0.62 (0.52–0.74)	<0.001 ***	−0.73	0.48 (0.37–0.62)	<0.001 ***	Male gender	0.63	1.87 (1.42–2.46)	<0.001***
Country (reference: Chile)							Country (reference: Chile)			
Denmark	−0.57	0.57 (0.38–0.84)	0.004**	1.59	4.46 (1.93–10.34)	<0.001***	Denmark	0.96	2.62 (1.55–4.43)	<0.001***
Netherlands	−0.79	0.46 (0.32–0.66)	<0.001***	1.71	5.55 (2.47–12.46)	<0.001***	Netherlands	-0.23	0.79 (0.43–1.45)	0.447 (ns)
Sweden	−0.63	0.53 (0.39–0.74)	<0.001***	2.25	9.44 (4.36–20.41)	<0.001***	Sweden	0.53	1.69 (1.08–2.65)	0.021*
PsA	0.20	1.22 (1.02–1.47)	0.03*	0.25	1.29 (1.03–1.61)	0.028*	PsA	0.39	1.47 (1.11–1.95)	0.007**
BSA (disease severity)	0.41	1.51 (1.26–1.82)	<0.001***	0.03	1.03 (0.94–1.13)	0.560 (ns)	Disease severity (reference: no disease/BSA=0)			
Constant	4.00	3.38	<0.001***	−3.13	0.04	<0.001***	BSA (less than 1 palm)	0.43	1.53 (.89–2.65)	0.125
							BSA (1–3 palms)	0.55	1.74 (1.01–3.00)	0.047*
							BSA (4–9 palms)	0.65	1.91 (1.08–3.38)	0.026*
							BSA (10–19 palms)	0.27	1.31 (.64–2.68)	0.467 (ns)
							BSA (20 palms or more)	0.87	2.38 (1.08–5.28)	0.032*
							Constant	-1.56		<0.001***

* P < 0.05,** P < 0.01, *** P < 0.001

BSA:Body Surface Area; 95%CI:95% confidence interval; OR:odds ratio; PsA:psoriatic arthritis.

## DISCUSSION

This international cross-sectional study expands current understanding of psoriasis triggers and disease dynamics highlighting heterogeneity of the disease experience. Although heterogeneous, reported triggers exhibit recurring patterns that enable classification and determination of their prevalence. Stress emerged as the most frequently reported trigger associated with flare-ups, along with other reported psychological factors, including life trauma/grief, depression and feelings of anxiety, all of which inherently involve psychological stress for affected individuals. Additionally, our findings demonstrated that self-reported disease triggers are highly prevalent, with 71% of participants identifying at least one trigger factor. Most participants experienced flare-ups or periods of increased psoriatic disease activity over the last 3 years, and the majority reported seasonal variation in their condition.

Infections, sleep deprivation, other diseases and medical interventions such as major surgeries may also impose stress on patients. Previously, increased daily stressors have been shown to correlate with subsequent increases in PASI scores (11), and controlled stress exposure in individuals with psoriasis (Trier Social Stress Test) (12) has been shown to alter immune functions related to psoriasis (13, 14). Recent data from the Swedish Military Service Conscription Register revealed that low stress resilience at conscription is associated with a higher risk of developing Pso and PsA later in life (15). This suggests that stress may not only trigger disease flare-ups, as reported by PSODEEP1 participants, but also contribute to disease onset. The number reporting stress as an important trigger factor (55.3%) aligns with a smaller questionnaire-based study (*n*=162), where 60.1% of participants reported stress as the most common disease trigger (16), 54.7% in the ActiPso study (*n*=586), and 68% in a report from ongoing UK-based mySkin (*n*=529) (17). Risk factors and triggers are arguably two separate entities; an overlap may be attributed to overlapping immunological mechanisms in disease onset and disease flare-up.

Although stress is the most frequently reported trigger among patients with psoriasis across populations, experimental research investigating the relationship between psychological stress and psoriasis pathophysiology remains limited. There is a need for more mechanistic studies on stress and psoriasis, since they could provide new pharmacological targets and offer new insights into the biology of early disease development. From an evolutionary perspective, upregulating the immune system to better handle skin injuries could be a favourable adaptation to threatening/stressful situations. This rationale is supported by the suggestion that the rate of wound healing is higher in psoriatic lesional skin than in non-lesional and healthy skin in control individuals (18).

Addressing stress could provide a path to achieving disease modification, as suggested by studies on psychological intervention, mindfulness and meditation for psoriasis, which demonstrated effects on both psoriasis skin activity and quality of life (19, 20). Several other triggers reported in this study, including alcohol consumption, diet, tobacco use and physical inactivity, can be addressed through lifestyle changes, although the impact of such changes on psoriasis disease activity needs further investigation. These triggers have previously been associated with psoriatic disease or the risk of psoriatic disease onset in various studies. Low cardiorespiratory fitness (21, 22) and weight gain are known risk factors for psoriasis onset (23). The Mediterranean diet can improve skin disease and diminish the need for topicals (24). Obesity is associated with worse treatment responses (25) and an increased risk of developing PsA (26). Lifestyle changes are challenging to address during brief outpatient consultations but may positively influence the risk of comorbidities, such as metabolic syndrome, cardiovascular disease and depression (27). Digital health strategies could provide cost-effective means to support lifestyle changes but need to show efficacy in scientific studies (28). Triggers reported with higher prevalence among individuals with PsA are conditions already shown to occur in higher frequency among individuals with PsA, such as obesity (26), sleeping disorders and depression (29). On the other hand, female participants did not report depression as a trigger more often than males, even though it is well established that the female sex implies a higher depression risk (30). Furthermore, female participants reported infections as a psoriatic disease trigger more often, although women have a lower risk than men to develop most infectious diseases. It has been suggested that women have fewer infections owing to a stronger innate immune response (31) and innate immune responses are central to the early steps in the development of new psoriasis lesions (32).

Cross-country differences in self-reported triggers of psoriasis may reflect broader cultural, societal, environmental or genetic differences. In the context of genetics, HLA-Cw*06 : 02, which is strongly associated with psoriasis susceptibility, has been associated with flare-ups triggered by throat infections (33). While the prevalence of HLA-Cw*06 : 02 among individuals with psoriasis in Chile is unknown, an earlier study on patients with PsA in South America (Colombia) demonstrated a remarkably low HLA-Cw*06 : 02 prevalence (34) when compared to studies from northern Europe, which could influence the occurrence of infection-triggered psoriasis.

### Limitations

This study had several limitations. It is natural for humans to create explanatory mental models for disease occurrence, potentially introducing a bias to identify causality or associations in temporally co-occurring random events. Whilst the open-question, exploratory approach resulted in the widest range of triggers described to date, the interpretation of free-text responses introduces a degree of subjectivity. The self-reported digital questionnaire format provided no means to ask clarifying follow-up questions and although self-reported flare-ups and disease activity estimation using BSA has been used before (35), these measures require further validation. Furthermore, complete trigger recall cannot be guaranteed, and the magnitude of the impact of individual triggers on disease activity was not assessed. The larger size of the Swedish cohort contributes more heavily to the pooled results, which may weaken the external validity of the findings. Despite these limitations, this study represents one of the largest international exploratory investigations of self-reported psoriasis triggers across diverse populations and healthcare systems.

### Conclusion

PSODEEP1 findings emphasize that patients frequently associate their disease activity with identifiable external or internal triggers. Future studies addressing whether removing triggers could lead to disease modification and prolonged periods of remission/lower disease are needed. The wide range of trigger subgroups provides a valuable foundation for both patient-physician dialogues and future trigger studies. Based on the patients” perspective, promoting overall health and psychological well-being in individuals with psoriasis is important, reinforcing the perception that psoriasis is a disease that requires both a holistic and individualized approach.

## Data Availability

The data that support the findings of this study are available from the corresponding author upon reasonable request.
