# Supplementary material for: Psoriasis Triggers and Disease Activity: Analysis of Survey Data from the PSODEEP1 Study
Source: Acta Derm Venereol. 2026 Jun 1;106:0167. doi: 10.2340/actadv.v106.adv-2025-0167 (PMC13224730; doi:10.2340/actadv.v106.adv-2025-0167)
Supplement: Supplementary Appendix [file ActaDv-106-0167-s0001.pdf]

PSODEEP1

Please complete the survey below.

Thank you!

Request to participate in the research project PsoDEEP<br><br>This is a digital survey that is distributed to patients with skin psoriasis and / or psoriatic arthritis. The aim is to increase the understanding of various aspects of the psoriasis disease such as perceived disease variation over time and factors that lead to disease deterioration. The survey takes about 15 minutes to complete.<br><br>Information is handled in accordance with current confidentiality regulations and the Data Protection Ordinance (GDPR) and stored on data servers belonging to XXXXX University. It will not be possible to identify you as a person during statistical data processing or when the study results are presented (scientific publications). Participation in the study is voluntary and will not have any consequences for your future care & treatment. You can withdraw your participation consent at any time by contacting the research nurse.	◯ I have read and understood the above information
By clicking accept, you give your consent to participate & you will proceed to the survey.	◯ Accept, take me on to the survey!<br>◯ Deny, I do not want to participate in PSODEEP.
Do you have any type of psoriasis or psoriatic arthritis?	◯ Yes, and I'm diagnosed by a physician<br>◯ Yes, but I'm not diagnosed by a physician<br>◯ I do not have any type of psoriasis<br>◯ Do not know
Which type of psoriasis have you been diagnosed with by a physician?	◯ Psoriasis on the skin, on nails or the scalp<br>◯ Psoriatic arthritis<br>◯ Both psoriasis (on skin, nails or scalp) and psoriatic arthritis<br>◯ Do not know
What is your age?	__________________________________
What gender are you?	◯ Woman<br>◯ Man<br>◯ Other alternative/do not wish to answer
When did you develop your first symptoms of psoriasis (on skin, nails or scalp)?	◯ Less than 1 year ago<br>◯ 1-2 years ago<br>◯ 3-5 years ago<br>◯ 6-15 years ago<br>◯ 16-25 years ago<br>◯ 25 years or more ago<br>◯ Don´t know

When did you develop your first symptoms of psoriasis arthritis?	◯ Less than 1 year ago<br>◯ 1-2 years ago<br>◯ 3-5 years ago<br>◯ 6-15 years ago<br>◯ 16-25 years ago<br>◯ 25 years or more ago<br>◯ Don´t know
If you think about the past 12 months, how would you rate the severity of your skin psoriasis ?	◯ Not severe at all<br>◯ Not particularly severe<br>◯ Quite severe<br>◯ Very severe<br>◯ Extremely severe<br>◯ Do not know
If you think about the past 12 months, how would you rate the severity of your psoriasis arthritis?	◯ Not severe at all<br>◯ Not particularly severe<br>◯ Quite severe<br>◯ Very severe<br>◯ Extremely severe<br>◯ Do not know
Based on the amount of skin psoriasis that could be covered by the palm of your hand, about how many palms would you say that you currently have across your entire body?	◯ None<br>◯ Less than 1 palm<br>◯ 1-3 palms<br>◯ 4-9 palms<br>◯ 10-19 palms<br>◯ 20 palms or more<br>◯ Do not know
How many of your joints are inflamed or painful right now?	◯ None<br>◯ Only 1<br>◯ 2 to 3<br>◯ 4 or more<br>◯ Do not know
Which statement/description suits your skin psoriasis the best?	◯ It is dominated by multiple wide spread small lesions (approx. centimeter in size) - eruptive psoriasis or guttate psoriasis<br>◯ It is dominated by larger confluent lesions - plaque psoriasis<br>◯ It is dominated by red/inflamed skin with yellow pustules - pustular psoriasis<br>◯ Do not know
How often have you had relapses or flare-ups of your psoriasis (on the skin, nails or scalp) in the past three years? With relapse or flare-up, we mean periods when symptoms have worsened.	◯ I´ve had constant symptoms, with no remission<br>◯ Every week-month<br>◯ Every quarter<br>◯ Every six months<br>◯ Every year<br>◯ Every two years<br>◯ Every three years (once in the past three years)<br>◯ I haven´t had any flare-up or relapses in the past three years<br>◯ Do not know
Do you experience seasonal variation in your psoriasis (e.g. with improvement in summer and worsening during winter)?	◯ Yes<br>◯ No<br>◯ Do not know

Have you experienced joint stiffness, pain, swelling or tenderness?	◯ Yes, right now<br>◯ Yes, in the last 12 months<br>◯ Yes, more than a year ago<br>◯ No, never<br>◯ Do not know
Which statement/description suits your experience of joint stiffness, pain, swelling or tenderness the best?	◯ Symptoms worsen after movement/exercise and are most pronounced during daytime (afternoon/evening)<br>◯ Symptoms improve after movement/exercise and are most pronounced during morning and/or during nighttime<br>◯ Do not know
Have you experienced pain or tenderness in muscle tendons?	◯ Yes, right now<br>◯ Yes, in the last 12 months<br>◯ Yes, more than a year ago<br>◯ No, never<br>◯ Do not know
Which statement/description suits your experience of pain or tenderness in muscle tendons the best?	◯ Symptoms worsen after movement/exercise and are most pronounced during daytime (afternoon/evening)<br>◯ Symptoms improve after movement/exercise and are most pronounced during morning and/or during nighttime<br>◯ Do not know
Have you ever experienced an entire finger or an entire toe swollen, like a sausage (dactylitis)?	◯ Yes<br>◯ No<br>◯ Do not know
Do your fingernails or toenails have holes or pits?	◯ Yes<br>◯ No<br>◯ Do not know
Have you ever had pain in your heel?	◯ Yes<br>◯ No<br>◯ Do not know
Does your skin psoriasis worsen due to scraping or scratching (e.g. secondary to itch)?	◯ Yes<br>◯ No<br>◯ Do not know
When does this happen? (worsening due to scraping or scratching)	◯ When I experience flare-up/periods of high disease activity (active disease)<br>◯ Constantly without variation over time<br>◯ It varies over time but is not associated to flare-ups (active disease)<br>◯ Don't know
Has your skin psoriasis ever developed at the site of a skin injury, such as a scrape or a burn?	◯ Yes<br>◯ No<br>◯ Do not know
When does this happen? (development of psoriasis due to skin injury)	◯ When I experience flare-up/periods of high disease activity (active disease)<br>◯ Constantly without variation over time<br>◯ It varies over time but is not associated to flare-ups (active disease)<br>◯ Don't know

Have you ever developed psoriatic arthritis in a joint after injury, e.g. after a fall or sprain?	◯ Yes<br>◯ No<br>◯ Do not know
When does this happen? (psoriatic arthritis development after injury)	◯ When I experience flare-up/periods of high disease activity (active disease)<br>◯ Constantly without variation over time<br>◯ It varies over time but is not associated to flare-ups (active disease)<br>◯ Don't know
Have you ever associated flare-ups/periods of increased disease activity with triggering factors (e.g. weight gain, stress, depressive mood, infections, medical drugs, alcohol, tobacco, sunburn or anything else)?	◯ Yes<br>◯ No<br>◯ Do not know
Please list factors that you personally associate with flare-ups/periods of increased disease activity.	__________________________________
Are you using any of the following treatments for your psoriasis/psoriatic arthritis?	☐ Emollients (such as creams or ointments without cortisone)<br>☐ Topical cortisone treatments (creams or ointments containing steroids)<br>☐ Alternative medicine (e.g. from health food stores)<br>☐ Phototherapy (e.g. UVB treatment)<br>☐ Oral (tablet) or injectable methotrexate<br>☐ Oral (tablet) medications (other than methotrexate)<br>☐ Injectable or intravenous biologic medication (infusions/injections/prefilled injection pen)<br>☐ Other treatment
Please provide your mobile phone number:	____________________________
Would you agree to be contacted by the research group if follow-up studies or new studies on psoriasis are perfomed?<br><br>If you select "YES" above then your contact information will be saved by the research group. If you select "NO" the mobile phone number you provided earlier will be erased during future data thinning procedure.	◯ Yes<br>◯ No
You have given the research group the possibility to contact you if follow-up or new studies on skin psoriasis/psoriatic arthritis are performed.<br>Please provide your email and city of residence (in addition to already provided mobile phone number).<br>Email:	____________________________
City of residence:	____________________________

In order to "submit" the study survey you first need to answer ALL questions.

Appendix S2. First 50 responses of each trigger subgroup.

A STRESS
1. Chile – Estres - Stress
2. DK – Stress - Stress
3. SWE – Stress - Stress
4. Chile- Estrés - Stress
5. NL – Stress - Stress
6. Chile - Estrés - Stress
7. NL – te vel stres brengt het in actie – too much stress leads to flare
8. DK – Stress – stress
9. Chile - Estrés - stress
10. Chile - Estrés - stress
11. DK- Stress- stress
12. Chile – Estrés- stress
13. NL- Stress- stress
14. Chile - estrés- stress
15. Chile – estres- stress
16. NL- Stress- stress
17. Chile - Estrés- stress
18. DK- Stress- stress
19. SWE- stress- stress
20. DK- stresset perioder- stressful periods
21. NL- stress- stress
22. SWE- stress- stress
23. DK- stress- stress
24. 12NL- stress- stress
25. SWE- stress- stress
26. Chile - estrés- stress
27. NL- stress- stress
28. SWE- stress- stress
29. Chile - estrés laboral y/o familiar- work and/or family stress
30. NL- stress van het werk- stress from work
31. DK- stress- stress
32. NL- stress- stress
33. SWE- stress- stress
34. Chile - estrés- stress
35. DK- stress- stress
36. SWE- stress-stress
37. Chile - estrés- stress
38. DK- belastning- load
39. NL- spannig- tension
40. SWE- stress- stress
41. Chile – En mi trabajo, una vez estuve con licencia por dos meses y mi psoriasis bajó considerablemente – In my work, once I was away from work two months and my psoriasis significantly improved
42. Chile – cuadro de estrés - stress episode
43. DK-stress-stress44. NL-stress, wekgerelateerd, ut huis gaan, onzekerheid- stress, wake-up related, leaving home, uncertainty
45. SWE- stress-stress
46. Chile – en el mes de marzo y abril, por mi actividad laboral..soy contador. In the months of march and april, due to my work, I am accountant.
47. DK- stress-stress
48. NL-stress- stress
49. DK- travlhed- busyness
50. Chile – estres -stress

B DEPRESSION
1. Chile - depresión - depression
2. Chile – estado de animo depresivo – depressive mood

3. SWE - nedstämdhet - depression
4. Chile - depresión -depression
5. DK – depression – depression
6. SWE - nedstämdhet - depression
7. SWE - nedstämdhet - depression
8. DK – depression - depression
9. DK – depression -depression
10. Chile – estado depresivo – depressive state
11. DK- depression- depression
12. Chile - depresión- depression
13. SWE-nedstämdhet- depression
14. Chile – estado de ánimo depresivo – depressive mood
15. DK- depression- depression
16. Chile - Depresión - depression
17. DK - depression- depression
18. DK -depression- depression
19. DK – depression- depression
20. Chile - depresión- depression
21. DK- tristhed- sadness
22. Chile – episodios depresivos - depressive episodes
23. SWE- nedstämdhet- depression
24. Chile – estado de animo depresivo- depressed mood
25. SWE- nedstämdhet- depression
26. DK- depression- depression
27. SWE – depression- depression
28. Chile - depresión- depression
29. SWE- nedstämdhet- depression
30. Chile - depresión - depression
31. Chile – estado de animo depresivo – depressive mood
32. DK- depression- depression
33. Chile – estado de animo depresivo – depressive mood
34. NL- ptss en depressie - ptsd and depression
35. SWE- nedstämdhet- depression36. Chile - depresión- depression
37. SWE – depression- depression
38. SWE- nedstämdhet- depression
39. DK- depression- depression
40. Chile - depresión- depression
41. DK- depression- depression
42. SWE- nedstämdhet- depression
43. Chile - depresión- depression
44. SWE- depression, depression
45. SWE- Nedstämdhet- depression
46. SWE- depression- depression
47. SWE- Nedstämdhet- depression
48. Chile – estado de animo depresivo - depressed state
49. SWE- depression- depression
50. SWE- depression (om man mår dåligt av något psykiskt) - depression (if you feel bad about something mentally)

C ANXIETY

1. SWE – oro - worry
2. Chile – ansiedad - anxiety
3. SWE - ångest - anxiety
4. Chile – ansiedad anxiety
5. DK – angst – anxiety
6. Chile – momentos de alta ansiedad – moments of high anxiety
7. SWE - ångest - anxiety
8. Chile – ansiedad, nervios – anxiety, nervousness
9. DK - nervøsitet - nervousness
10. SWE – oro – worry

11. Chile – crisis ansiedad – panic attack
12. SWE- oro- worry
13. DK- Nervøsitet og stress- Nervousness and stress.
14. DK- uoplagthed- uncertainty
15. NL- stress of zorgen- stress or worry
16. Chile – ansiedad - anxiety
17. Chile – ansiedad - anxiety
18. SWE- vid flygskräck- in case of fear of flying
19. Chile – mayor ansiedad – mayor worry
20. Chile – ansiedad/angustía/preocupación - anxiety
21. Chile – ansiedad – anxiety
22. Chile – ansiedad - anxiety
23. Chile – nervios - nerves
24. SWE- oro- worry
25. SWE- ångest och oro, anxiety and worry
26. Chile – ansiedad - anxiety
27. Chile – ansiedad - anxiety
28. SWE- ångest - anxiety29. Chile – preocupaciones varias - various worries
30. SWE- ångest- anxiety
31. Chile – preocupaciones - worries
32. Chile – ansiedad – anxiety
33. Chile – ansiedad – anxiety
34. Chile – post cuadros de ansiedad - after panic attacks
35. Chile – ansiedad - anxiety
36. Chile – ansiedad - anxiety
37. Chile – ansiedad – anxiety
38. Chile – ansiedad episodica – panic attacks
39. SWE- vid oro- worriness
40. Chile – preocupaciones - worries
41. Chile – ansiedad, preocupación - anxiety, worry
42. DK- Har døget med angst og stress, og der var der meget slemt. Får det mest når jeg er presset. - I've dealt with anxiety and stress, and it was very bad. I get it most when I'm under pressure.
43. Chile – ansiedad - anxiety
44. Chile – ansiedad - anxiety
45. Chile – ansiedad - anxiety
46. NL- zenuwachtig- nervous
47. SWE - Hudpsoriasisen ser jag en säker koppling till stress och oro. - I see a definite link between skin psoriasis and stress and anxiety.
48. SWE- oro- worry
49. SWE- Ångest- anxiety
50. SWE- oro- worry

D OTHER PSYCHOLOGICAL
1. SWE – starka känslor (positiva och negativa) - strong emotions (positive and negative)
2. DK – ude af balance humørmæssigt - mood out of balance
3. Chile – estado de ánimo - mood
4. NL – uit huis gaan, onzekerheid – moving out from parental home, insecurity
5. Chile – estado de animo – mood
6. Chile – estado de ánimo - mood
7. Chile – no desahogar la rabia – not to vent anger
8. SWE – psykiskt mående - mental health
9. DK - dårligt humør - bad temper
10. DK - psykiskt tilstand – mental state
11. DK- ked af det - feeling sad
12. Chile – estado de ánimo- mood
13. DK- psykisk uligevægt - mentally unbalanced
14. Chile – estado de animo - mood
15. Chile – estado de animo - mood

16. Chile – estado de ánimo - mood
17. Chile – hostigamiento laboral – workplace harassment
18. Chile – cambios de humor (enojo) - mood swings (anger)19. DK- psykisk ustablitet- mentally unbalanced
20. Chile – estado de ánimo - mood
21. SWE- otrivsel- not thriving
22. Chile – estados de ánimo - mood
23. NL- ptss-PTSD
24. Chile – estado de ánimo - mood
25. NL- ptss- PTSD
26. NL- automutilatie door post traumatische stress stoornis inclusief de ptss onstaan bij kinderen van 6 jaar en jonger - self-harm due to post traumatic stress disorder including ptsd from when I was 6 years and younger
27. Chile – estado anímico - mood
28. DK- mentale problemer- mental problems
29. Chile – desequilibrios del ánimo, irresponsabilidad emocional – mood inbalances, emotional irresponsabilities
30. DK- Stress, følelsesmæssig ubalance. Men ikke altid.- stress, emotional imbalance. But not always.
31. Chile – estados de ánimo - mood
32. SWE- psykisk ohälsa leder ofta till värre psoriasis utbrott. - mental health problems often lead to worse psoriasis outbreaks.
33. Chile – estado de ánimo - mood
34. Chile – estado de animo - mood
35. SWE- När jag mår dåligt. - When I am not feeling good.
36. Chile – estado de ánimo - mood
37. Chile – estado de ánimo - mood
38. Chile – estado de ánimo - mood
39. Chile – estado de ánimo - mood
40. DK- blive ked af det, at være ked af det uden nogen rigtig ground, getting upset, being upset without any real reason
41. DK- psyke- psyche
42. SWE- upprördhet- being upset
43. Chile - tención emocional – emotional tension
44. Chile – enojo - anger
45. Chile – mal genio – bad temper
46. DK- stress- anden psykisk påvirkning- stress- other psychological impact
47. DK- Min ADD/ Stress - My ADD/ Stress
48. SWE- Psykiskt mående- mental health
49. SWE- Psykisk hälsa- mental health
50. NL- verschillende emoties (vedriet, boosheid, wondjes)- various emotions (sadness, anger, wounds)

E LIFE TRAUMA/GRIEF
1. DK – i forbindelse med nær slægtninge død - in connection to death of close relatives
2. DK – sorg – sorrow
3. SWE – negativa händelser - negative events
4. Chile – rupturas amorosas – breakups
5. DK - sorg - sorrow
6. DK – sorg – sorrow
7. Chile – penas – sorrow
8. DK – sorg – sorrow
9. DK - dødfald i familien – death in the family
10. DK – skilsmisse – divorce
11. Chile – pena por muerte de familiares – sorrow caused by death of relatives
12. SWE- trauma- trauma
13. NL- ooit begonnen 60 jaar geleden verhuizing, en vaker rond verhuizingen, stressituaties, overlijdens oon 4 jaar geleden. - Started 60 years ago when I moved, and more often around moving, stressful situations, deaths 4 years ago.
14. DK- traume- trauma

15. Chile – perdidas emocionales, conflictos laborales – emotional losses, labour conflicts
16. SWE- Vid nära anhörigs fleråriga sjukdomsperiod och dödsfall kom nya fläckar. - The illness and death of a close relative over a period of several years resulted in new patches.
17. DK- Da min mor blev erklæret terminal. - When my mother was declared terminal.
18. SWE- Närstående diagnostiserad med cancer. - Relative diagnosed with cancer.
19. DK- traume- trauma
20. SWE- sorg- sorrow
21. Chile – traumas físicos y emocionales – emotional and physical traumas
22. SWE- Min makes sjukdom och död. - My husband's illness and death.
23. DK- Stress og trauma oplevelser såsom chok eller ekstrem belastning. Job stress over flere måneder f.eks. eller uventet oplevelser såsom et voldsomt skænderi. - Stress and traumatic experiences such as shock or extreme stress. Job stress over several months, for example, or unexpected experiences such as a violent injury.
24. NL- sterfgevallen- deaths
25. NL- stress ivm overlijden ouders- stress due to death of parents
26. SWE- Stress, ex anhörigas död- Stress, e.g. death of a relative.
27. NL- na veel stress op bv werk/overlijden in de familie.- after a lot of stress e.g. at work/death in the family.
28. NL- stress bij de hartaanval van mijn man en het overlijden van mijn schoonmoeder is het gestart op mijn handpalmen hoofdhuid begon heel erg the vlammen en in mijn grote teenagels zat het er helemaal in- stress after my husband's heart attack and the death of my mother-in-law it started on my palms scalp started very much the flame and in my big toe nails it was all over the place
29. SWE- Psykisk stress p.g.a. dödsfall i familjen m.m.- Psychological stress due to death in the family, etc.
30. NL- scheiding- divorce
31. NL- nadat ik angina heb gehad en bij veel stress- after I had angina and when under a lot of stress
32. SWE- sjukdom och dödsfall- illness and death
33. NL- overlijden van een dierbaar persoon- death of a loved one34. NL- spanningen door ernstige ziekte van ouders- tensions due to serious illness of parents
35. SWE- Vid stress då min bästa vän dog. Då fick jag helt nya fläckar och områden. Hade tidigare bara i hårbotten och i och runt öronen. - Under stress when my best friend died. Then I got completely new spots and areas. Had previously only on the scalp and in and around the ears.
36. SWE- skilmässa på 1980-talet- divorce in the 1980s.
37. SWE- ledsamheter- sorrows
38. SWE- sorg- sorrow
39. SWE- sorg- sorrow
40. SWE- “ledsamheter” i omgivningen- “sorrows” in the surroundings
41. SWE- trauma- trauma
42. SWE- i samband med en arbetsplatsolycka där många aktörer som försäkringskassan, företaget, facket som tecknad arbetsskadeförsäkring plus då i förlängningen afa, försäkringsbolaget som företaget var knutet till och andra försäkringar kopplat till arbetsplatsolyckan. Allt detta är/var utlösande faktorer. Trauma efter olycksfallet. - in connection with a workplace accident where many actors such as the social insurance office, the company, the trade union that took out work injury insurance plus then by extension afa, the insurance company to which the company was linked and other insurance linked to the workplace accident. All these are/were triggering factors. Trauma after the accident.
43. SWE- sjukdom hos en närstående- illness of a close relative
44. SWE- skilsmässa- divorce
45. SWE- sorg- sorrow
46. SWE- Min hustru fick diagnos Alzheimer 2018. Det var därefter som jag fick mina

första fläckar efteråt.- My wife was diagnosed with Alzheimer's in 2018. It was afterwards that I got my first patches.
47. SWE- sorg- sorrow
48. SWE- sorg- sorrow
49. SWE- personligt trauma i samband med dödsfall i min närhet. - personal trauma related to deaths in my surroundings.
50. SWE- Stress (särskilt känslomässig, som skilsmässa, sorg)- Stress (especially emotional, like divorce, grief)

F INFECTIONS
1. NL- een infectie- an infection
2. DK- infektioner- infections
3. DK- infektioner- infections
4. NL – na infectie (bv griep) - after infection (e.g. flu)
5. SWE – infektioner – infections
6. NL –infectie- infections
7. SWE – infektioner – infections
8. SWE – infektion – infection
9. NL - stress, zowel lichamelijk (bijvoorbeeld virus) als ook emotioneel. - stress, both physical (e.g. virus) and emotional10. NL- bij infecties zoals streptokokken - for infections such as streptococcus.
11. NL- keelontsteking week erna zat ik helemaal onder tot 2x toe- laryngitis week after I was completely covered up to twice
12. DK- infektioner- infections
13. SWE- Infektion- Infection
14. NL- Stress en infecties. Als ik bijboorbeeld keelontsteking heb is mijn psoriasis echt aanzienlijk veel erger (roder en veel meer jeuk). Stress and infections. When I have strep throat, for example, my psoriasis is really significantly worse (redder and much more itchy).
15. NL- na infecties- after infections.
16. SWE- infektion- infection
17. DK- infektioner- infections
18. SWE- vid infektioner i kroppen.- infections in the body.
19. DK- infektion- infection
20. DK- infektioner- infections
21. SWE- infektioner- infections
22. Chile – infecciones- infections
23. DK- infektioner- infections
24. Chile – cuando me enfermo respiratoriamente- when I get respiratory sickness
25. DK- infektioner- infections
26. SWE- covid och halsfluss- covid and tonsillitis
27. NL- griep- influenza
28. SWE- virussjukdomar- viral infections
29. SWE- förkylning- cold
30. Chile – infecciones en la piel - infections in the skin
31. DK- infektioner- infections
32. Chile - infección urinaria, amigdalitis – urinary tract infections, tonsillitis
33. NL- na ziekte aan de luchtwegen- after respiratory illness
34. DK- Halsbetændelse- tonsillitis
35. NL- infecties, sepsis- infections, sepsis
36. SWE- infektioner- infections
37. DK- infektion (kommer dage/uge efter)- infection (comes days/weeks later)
38. DK –influenza- influenza
39. DK- halsbetændelse(strepA)- tonsillitis(strepA)
40. SWE- Förkylningar- Colds
41. NL- keelontsteking- laryngitis
42. SWE- infektion- infection
43. SWE- halsfluss- tonsillitis
44. NL- Door keelonsteking- After laryngitis
45. chile – resfrío - cold
46. DK- Mindre infektioner. - Smaller infections.

47. DK- sygdom/infektioner- illness/infections
48. SWE- Förkylning- Cold
49. DK- Infektioner, selv de mindste som hurtigt er ovre og ikke behøver lægehjaelp. - Infections, even the smallest ones that are quickly resolved and do not require medical attention.
50. SWE- förkylning- cold

G DRUGS
1. Chile – medicamento – medication
2. SWE - läkemedel - drugs
3. NL- reactie op bepaalde medicijnen- reaction to certain medications
4. NL - en ik denk zelf bij gebruik van mijn medicijn ibertesan- and I think myself when using my drug ibertesan
5. NL – medicijngebruik- drug use
6. DK - medicin- drugs
7. Chile – drogas – drugs
8. Chile – medicamentos – medications
9. Chile – medicamentos - medications
10. DK – medicin- drugs
11. NL- na antibioticakuur- after a course of antibiotics
12. SWE – cytostatika behandling paxlitakel – cytostatic treatment (Paklitaxel)
13. NL- psoriasis is ontstaan samen met medicatie voor langzame scildklier- psoriasis arose along with medication for slow thyroid gland
14. Chile – medicamento litio - litium medication
15. DK- Indtagelse af medicin.- Taking medication.
16. NL- Sinds het slikken van bloedverdunners is de psoriasis verergerd. - Since taking blood thinners, psoriasis has worsened.
17. SWE - läkemedel - drugs
18. NL- mediactie- drugs
19. NL- gebruik van pijnstillers zoals Brufen 800(via de reumatoloog)- use of painkillers such as Brufen 800(through the rheumatologist)
20. NL- geneesmiddelen- drugs
21. SWE – mediciner för adhd – medications for adhd
22. Chile – medicamentos – medications
23. NL- Prednison- Prednisone
24. SWE - läkemedel - drugs
25. NL- medicatie- drugs
26. SWE – efter cellgiftsbehandling, allt gick bort sen kom det tillbaka med förnyad kraft och värre än innan – after chemotherpy, everything disapeared but did then come back with new force and much worse than before
27. NL- medicatie- drugs
28. NL- Ibuprofen- Ibuprofen
29. NL- Pijnstillers (na een breuk)- Painkillers (after a fracture)
30. SWE - läkemedel - drugs
31. NL- geneesmiddelen- drugs
32. DK- ny medicin- new drug
33. NL- Een antibiotica genomen en daarna begin het de eerste keer- Took an antibiotic and then started it the first time
34. SWE – penicillin – penicillin
35. DK- Medicin- drug
36. NL- geneesmiddelen- drugs
37. SWE – vissa blodtrycksmediciner – certain anti-hypertensive drugs38. NL- antidepressiva- antidepressants
39. NL- medicatie: Zaralto zeer snel toename psoriasis Metoprolol geeft ook toename en lastiger te behandelen psoriasis- medication: Zaralto very fast increase in psoriasis Metoprolol also gives increase and more difficult to treat psoriasis
40. SWE - möjligen läkemedel - possibly drugs
41. SWE – en del läkemedel - some types of drugs
42. NL- medicijnen- drugs
43. NL- antibiotica – antibiotics

44. NL- geneesmiddelen- drugs
45. NL- medicatie- drugs
46. NL- medicatie- drugs
47. NL- sommige geneesmiddelen- some drugs
48. NL- tijdens en na rcd kuren- during and after rcd cures
49. NL- geneesmiddelen- drugs
50. NL- Psoriasis verergerde bij gebruik van bepaalde medicijnen- Psoriasis worsened when taking certain drugs

H HORMONAL CHANGES
1. Chile- cambio anticonceptivos (pastillas) - change of pregnancy control (pills)
2. NL – cyclus- menstrual cycle
3. DK – Hormonelt betinget Brød ud efter første graviditet for 30 år siden og igen i forbindelse med overgansalder- Hormone-related Broke out after first pregnancy 30 years ago and again during menopause
4. DK- Min menstruationscyklus- My menstrual cycle
5. DK – Graviditet og amning – Pregnancy and breastfeeding
6. DK - præventions piller med hormon- contraceptive pills with hormones
7. SWE – efter graviditet – after pregnancy
8. SWE – hormonella förändringar graviditet/förlossning - hormonal changes pregnancy/childbirth
9. SWE – hormonella förändringar - hormonal changes
10. NL – Uitbraak na zwangerschappen ook- Outbreak after pregnancies too
11. SWE – graviditet – pregnancy
12. SWE – hormoner – hormones
13. DK- hurmoner- hormones
14. DK- Menstruation (det bliver typisk værre ugen op till menstruation, hver måned)- Menstruation (it typically gets worse the week before menstruation, every month)
15. NL- Na zwangerschappen (tijdens zwangerschappen juist geen last)- After pregnancies (during pregnancies no problems at all)
16. Chile - menstruación - menstruation
17. DK- Cyklus (hormoner)- cycle (hormones)
18. DK- jeg synes overgangsalderen har forvaerret både min gigt og min psoriasis og min feber- I think menopause has aggravated both my arthritis and my psoriasis and my fever
19. SWE – hormonell obalans – hormonal inbalances20. DK- amning (siden jeg fødte min anden søn har min psoriasis blevet meget forværret, modsat slog graviditeten den ned)- breastfeeding (since giving birth to my second son, my psoriasis has gotten much worse, whereas pregnancy knocked it down)
21. SWE – graviditet och amning – pregnancy and breastfeeding
22. NL- hormonale schommelingen- hormonal fluctuations
23. NL- hormonen, enorme verergering na de zwangerschappen- hormones, massive exacerbation after pregnancies
24. NL- Ook na de bevalling van mijn eerste kind een erge uitbraak gekregen- Also got a severe outbreak after giving birth to my first child
25. NL- menstruatie ik heb endometriose- menstruation I have endometriosis
26. NL- hormoon schommelingen- hormone fluctuations
27. NL menstruatie cyclus- menstrual cycle
28. DK- og desuden er den starkt forværret efter menopause gik igang- and in addition, it is strongly aggravated after menopause started
29. DK- Min psoriasis er først rigtig kommet voldsomt frem efter mine graviditeter- My psoriasis only really came out in full force after my pregnancies
30. DK-hormoner- især ifm menstruation- Hormones - especially during menstruation
31. NL- hormonaal- hormonal
32. SWE – mens perioder/klimakteriet - menstruation periods/menopause
33. NL- overgang- menopause
34. NL- tijdens mijn menstruatiecycles: tussen eisprong en menstruatie opvlamming. - During my menstrual cycles: between ovulation and menstrual flare-up.

35. SWE – menstruation – menstruation
36. SWE – hormoner dvs puberteten, efter graviditet, klimakteriet – hormones i.e. puberty, after pregnancy, menopause
37. SWE – menstruation
38. SWE - båda tillfällen när jag fick barn och ammade – both times I gave birth and breastfed
39. SWE – hormonellt, vid graviditet – hormones, during pregnancy
40. SWE – hormonella förändringar (pubertet, graviditet) - hormonal changes (puberty, pregnancy)
41. SWE – Amning – breastfeeding
42. SWE – blossade upp agressivt när jag blev mamma vid 21 års ålder - aggressive flare when I beacame a mother at the age of 21
43. SWE - när jag var gravid – when I was pregnant
44. SWE – menstruation
45. SWE – under graviditeten med vårt yngsta barn (flicka) - during the pregnancy of our youngest child (girl)
46. SWE – hormoner – hormones
47. SWE – pubertet och klimakteriet – puberty and menopause
48. SWE – klimakteriet fick sjukdomen att eskalera ohejdat – menopause made the disease escalate unheeded
49. SWE – efter att jag fött barn – after giving birth
50. SWE - barnafödande - giving birth

I MEDICAL CONDITIONS/ OTHER DISEASE UNSPECIFIED
1. DK – ved sygdom - in case of illness
2. DK sygdom- disease
3. NK- na ziekte – after illness
4. DK - mangel på d-vitamin - Vitamin D deficiency
5. NL –ziekte- illness
6. DK – sygdom- disease
7. DK – alm sygdom- common illness
8. SWE – sjuka – sickness
9. DK- sygdom- disease
10. SWE – sjukdomar – sicknesses
11. DK- sygdomme- sicknesses
12. DK- sygdom- sickness
13. DK- Kronisk nældefeber - Chronic urticaria
14. DK- sygdom-sickness
15. Chile – fibromialgia – fibromyalgia
16. Chile – tiroide regularizada - thyroide disease
17. SWE – sjukdom – sickness
18. SWE – sickness
19. Chile – brote de colitis ulcerosa – flare in ulcerative colitis
20. SWE – sjukdom – sickness
21. NL- ziekte- sickness
22. DK- Sygdom- sickness
23. DK- ved sygdom-during sickness
24. NL- slechte schildklier- bad thyroid
25. NL- ziek zijn- being sick
26. DK- alm sygdom- general sickness.
27. Chile – reacciones alérgicas - allergic reactions
28. DK- sygdom- sickness
29. DK- sygdom- sickness
30. DK- sygdom- sickness
31. NL- ziekte- sickness
32. DK- ved sygdom- during sickness
33. DK- sygdom- sickness
34. NL- ziekte- sickness
35. SWE – glutenintolerans – gluten intolerance
36. NL- ziekte- sickness

37. NL- ziekte- sickness
38. SWE – diabetes 2 - diabetes type II
39. SWE – sjukdom - sickness
40. NL- bij verminderde weerstand door ziekte- in case of reduced immunity due to sickness
41. SWE – tandproblem – toothproblems
42. SWE – annan sjukdom – other sicknesses
43. SWE – fibromyalgi – fibromyalgia44. SWE – sjukdom – sickness
45. SWE – sjuk – sick
46. SWE - högt blodsocker – high blood sugar
47. SWE – vid sjukdom – when illness occurs
48. SWE – sjukdom – sickness
49. SWE – efter cushing – after cushings
50. SWE – sjukdom – sickness

J SYMPTOMS OF DISEASE/MALAISE
1. NL- conditie- condition
2. SWE - hård mage – constipation
3. NL – verlaagde weerstand- lowered resistance
4. NL- pijn – pain
5. NL- onstekingen in lichaam- inflammations in body
6. NL- vermoeidheid- fatigue
7. NL- terug gang van de weerstand door ziek zijn- decline in resistance due to illness
8. NL- ontsteking in het lichaam- inflammation in the body
9. NL lage weerstand- low resistance
10. SWE – inflammation i kroppen – inflammation in the body
11. NL- vermindere weerstand- lowered resistance
12. SWE - dålig hälsa - bad health
13. DK- nedsat immunfunktion- impaired immune function
14. DK- træthed- tiredness
15. SWE - trötthet - tiredness
16. DK- træthed-tiredness
17. NL- vermoeidheid- fatigue
18. SWE - värk - pain
19. NL- Niet lekker in mijn vel zitten, niet fit zijn. - Not being comfortable in my skin, not being fit.
20. SWE - när magen krånglar - when stomach is upset
21. NL- Bij verminderde weerstand door ziekte. - In case of reduced immunity due to illness.
22. NL- en verlaagde weerstand- lowered immunity.
23. SWE - trötthet - tiredness
24. SWE – tarminflammation – gut inflammation
25. SWE - trötthet - tiredness
26. SWE – magproblem – stomach problems
27. SWE - trötthet - tiredness
28. SWE – tarm besvär - gut problems
29. SWE – magproblem – stomach problems
30. SWE - trötthet - tiredness
31. SWE - trötthet - tiredness
32. SWE – magproblem – stomach problems
33. SWE – gallbesvär/magkatarr – biledisorders/gastritis
34. SWE - smärta - pain35. SWE - smärta - pain
36. SWE – magproblem – stomach problems
37. SWE - diarré som inte är orsakad av magsjuka – diarrhea not caused by gastroenteritis
38. SWE - trötthet - tiredness
39. SWE - värk – pain

K MEDICAL PROCEDURES

1. SWE – efter min hjärtoperation - after my heart surgery
2. DK- Jeg har indtil for 1 år siden ikke haft psoriasis, men så blev jeg opereret for brystkræft og efter det fik jeg psoriasis over hele kroppen. Går i lysbehandling nu hos en hudlæge. - I didn't have psoriasis until a year ago, but then I had surgery for breast cancer and after that I got psoriasis all over my body. I am now undergoing phototherapy with a dermatologist.
3. DK- Vægttab på 35 kg efter en gastric sleeve operation. - Weight loss of 35kg after gastric sleeve surgery.
4. NL- na operatie- after surgery
5. DK- Som 73årig begyndte traumerne, og efter et par operation, underliv og kirtler bag skjoldbrusskirteln, kom psoriasisgigten for alvor. - At 73, the trauma began, and after a couple of operations, abdomen and glands behind the thyroid, the psoriatic arthritis really kicked in.
6. DK – operation- surgery
7. NL - ik ben anderhalf jaar geleden geoperereerd en is er een bijnier verwijderd. Had slecht een paar kleine plekjes en sindsdien is het geexplodeerd.- I had surgery a year and a half ago and an adrenal gland was removed. Had only a few small spots and since then it has exploded.
8. SWE – operationer – surgeries
9. SWE – rotfyllning av tand – dental root canal tratment
10. DK- operation- surgery
11. NL – operatie - surgery
12. SWE – operation – surgery
13. SWE – operationer – surgeries
14. SWE – operation – surgery
15. SWE – operation – surgery
16. SWE – operation av artos i höfled - surgery because of hip arthrosis
17. SWE – magproblem – stomach problems
18. SWE - efterfølgende operation – after surgery

L VACCINATIONS
1. NL- Ook mijn 2e corona vaccinatie had flinke invloed.- My 2nd corona vaccination also had quite an impact.
2. Chile – la primera dosis de vacuna China contra COVID – the first dose of the Chinese COVID vaccine
3. NL- vaccinatie van de corona- corona vaccination
4. NL- na de corona prik – after the corona vaccination
5. SWE – vaccin - vaccine
6. SWE – Covidvaccination nummer 2 – second COVID vaccine
7. SWE – Kanske covidvaccin? - maybe covid vaccine?
8. SWE – Covid vaccinationer – COVID Vaccinations
9. SWE – Covid-vaccin – COVID vaccine
10. SWE – Covidvaccin – COVID vaccine
11. SWE – efter fjärde covidsprutan då jag fick Modernas vaccin i juni – after the fourth covid shot when I got Modernas' vaccine in June
12. SWE – covidvaccin – COVID vaccine
13. SWE – vaccin mot covid 19 – vaccine against COVID 19
14. SWE – corona vaccinet – Corona vaccine
15. SWE – vaccinering mot covid – vaccination against COVID
16. SWE - vaccin – vaccination

M ALCOHOL
1. DK - alkohol- alcohol
2. NL –alcohol- alcohol
3. SWE – alkohol (mest starköl) - alcohol (mostly beer with higher alcohol content)
4. DK – alkohol- alcohol
5. DK – alkoholindtag- alcohol intake
6. SWE – alkohol – alcohol
7. DK- alkohol- alcohol
8. NL – gebruik alcohol- alcohol consumption

9. DK- alkohol- alcohol
10. NL- alcohol- alcohol
11. DK- indtag af alkohol- intake of alcohol
12. DK- alkohol- alcohol
13. Chile – Alcohol
14. SWE – alkohol – alcohol
15. Chile – alcohol
16. Chile – alcohol
17. SWE – alkohol - alcohol
18. SWE – alkohol- alcohol
19. DK- Alkohol- alcohol
20. NL- Feestdagen of verjaardagen waar je wat anders eet en wat meer alcohol nuttigt- Holidays or birthdays where you eat a little differently and consume a bit more alcohol.
21. NL- alcohol
22. SWE – alkohol
23. Chile – Alcohol
24. DK- Alkohol- alcohol
25. NL- Alcohol
26. NL drank- drinking
27. Chile – alcohol
28. DK- alkohol- alcohol
29. NL – alcohol - alcohol30. DK- alkohol-alcohol
31. DK- alkohol- alcohol
32. Chile – alcohol
33. SWE – alkohol – alcohol
34. SWE – alkohol – alcohol
35. SWE – alkohol – alcohol
36. DK- alkohol- alcohol
37. SWE – alkohol – alcohol
38. NL- alcohol
39. Chile –alcohol
40. DK- alkohol- alcohol
41. Chile – alcohol
42. NL- alcohol
43. DK- Alkohol- alcohol
44. DK- alkohol- alcohol
45. DK- alkohol- alcohol
46. Chile –alcohol
47. Chile – alcohol
48. Chile – Alcohol
49. DK- alkohol- alcohol
50. DK - alkohol –alcohol

N DIET
1. DK- farvestoffer i maden, sukker og hvidt brod- food colouring, sugar and white bread
2. Chile – mala alimentación - poor nutrition
3. NL- soms ook wel gefrituurd eten- sometimes fried food
4. NL - reactie op bepaalde voedingsstoffen- reaction to certain nutrients
5. SWE – socker – sugar
6. DK – kost-diet
7. Chile – mala alimentación - poor nutrition
8. DK- øget sukker indtag, dårlig kost- increased sugar intake, poor diet
9. SWE - dålig kost – poor diet
10. NL- het eten van veel suiker- eating a lot of sugar
11. NL- bepaalde voeding- certain food
12. NL- Feestdagen of verjaardagen waar je wat anders eet en wat meer alcohol nuttigt- Holidays or birthdays where you eat a little differently and consume a bit more alcohol.

13. DK- ved indtag af meget usund mad- when consuming very unhealthy food
14. SWE – lax, sur dricka, citron, tomat, konserveringsmedel/matburk, ägg, cola – salmon, acidic drinks, lemon, tomato, preservatives/canned food, egg, coke
15. DK- sukker- sugar
16. NL- bepaalde voedingsmiddelen (gluten, suiker, ws ook bij koemelk/lactose)- certain foods (gluten, sugar, probably also cow's milk/lactose)
17. NL- varkensvlees- pork
18. NL- eten van varkensvlees- eating pork
19. DK- dårlig kost- bad diet
20. SWE - dålig kost – bad diet
21. DK- dårlig kost- bad diet
22. NL- bepaalde voedsel producten zoals nachtschades- certain food products such as nightshades
23. SWE – gluten
24. Chile – alimientos aji - foods with chili pepper
25. Chile – exceso de azúcar - excess sugar
26. SWE – gluten
27. SWE – socker/snabbmat - sugar/fast food
28. DK- kosten- diet
29. NL- voeding- food
30. Chile – consumo de chocolate consumo de producto con gluten – choclate and gluten containing products
31. NL- chocolade- chokolate
32. DK- for meget slik og usundt mad- too much sweets and unhealthy food
33. DK- Matvarer. Krydder, kullsyreholdig drikke.
- Food products. Spices, carbonated drinks.
34. NL- veel suiker eten- eating a lot of sugar
35. DK- Sukkerstroffer og frøolier er udfaset. Har det bedre end i mange år!- Sugar syrups and seed oils have been phased out. Feeling better than I have in years!
36. SWE – gluten
37. DK- Indtagelse af animalsk fedt, sukker og alkohol- Intake of animal fats, sugar and alcohol
38. DK- For meget sukker fra slik- Too much sugar from candy
39. Chile – mala alimentacion – poor diet
40. DK- oksekød, svinekød, mælkeprodukter- beef, pork, dairy products
41. DK- noget mad- some food.
42. chile – comidas con ají - foods with chili pepper
43. SWE – socker – sugar
44. DK- Kost- Diet
45. Chile – comidas irritantes en general - triggering foods in general
46. Chile – cuando como harinas, cuando también consumo lácteos - flour, diary products
47. DK- For meget sukker, hvidløg- Too much sugar, garlic
48. NL- ‘slecht’ ongezond eten: veel vetten en suikers- 'bad' unhealthy food: lots of fats and sugars
49. DK- indtag af: alkohol, hurtige kulhydrater såsom sukker, hvidt brød, pasta mm svine- og oksekød- intake of: alcohol, fast carbohydrates such as sugar, white bread, pasta, pork and beef
50. Chile – aumento en el consumo de carbohidratos – increased consumption of carbohydrates

O WEIGHT GAIN

1. Chile – aumento de peso – weight gain2. SWE – jag har uppfattat det som att jag blivit bättre i min psoriasis när jag gått ner i vikt (från lite övervikt till normal) - I have perceived that my psoriasis has improved when I have lost weight (from a bit overweight to normal)
3. Chile – aumento de peso – weight gain
4. SWE - viktuppgång - weight gain
5. DK - vægt- weight
6. Chile – aumento de peso – weight gain
7. Chile – aumento de peso - weight gain

8. DK- vægtøgning - weight gain
9. Chile – aumento de peso - weight gain
10. DK- vægt- weight
11. Chile – aumento de peso – weight gain
12. Chile –sobre peso – overweight
13. Chile – peso – weight
14. Chile – aumento de peso – weight gain
15. NL- gewichtstoename- weight gain
16. Chile – Aumento de peso – weight gain
17. Chile – peso – weight
18. DK- Vægtøgning- weight gain
19. SWE - viktuppgång - weight gain
20. Chile – Aumento de peso – weight gain
21. Chile – aumento de peso – weight gain
22. SWE – weight gain
23. DK- overvægt- overweight
24. SWE – vikt – weight
25. Chile – Peso – weight
26. NL- periodes van dikker worden - periods of getting fatter
27. Chile – Aumento de peso – weight gain
28. SWE - viktuppgång - weight gain
29. Chile – sobrepeso – overweight
30. Chile – sobre peso – overweight
31. DK- Jeg antager også vægtøgning- I also assume weight gain
32. Chile – Aumento de peso – weight gain
33. DK- Hvis jeg tager på, bliver det også værre (har bmi på 21,5)- If I gain weight, it also gets worse (my bmi is 21.5)
34. Chile – Aumento de peso – weight gain
35. Chile – Con aumento de peso – when weight increases
36. DK- vægtøgning- weight gain
37. Chile – Aumento de peso – weight gain
38. SWE - viktuppgång - weight gain
39. Chile - aumentó de peso – weight gain
40. DK- vægtøgning- weight gain
41. Chile – obesidad – obesity
42. Chile – aumento de peso – weight gain
43. DK - vægt- weight
44. SWE - viktuppgång - weight gain
45. SWE – viktuppgång - weight gain46. Chile - Aumento de peso – weight gain
47. SWE - viktuppgång - weight gain
48. Chile – sobrepeso – overweight
49. NL- overgewicht- overweight
50. DK- vægt- weight

P PHYSICAL EXERTION
1. DK – og ved overanstrengelse - and in case of overexertion
2. DK- vægtløftning- weightlifting
3. Chile – mucho rato escribiendo a computador
4. DK- overbelastning fx at løfte for tungt ensidige bevægelser i for lang tid fx køretur / vunduevask /støvsugning- overloading e.g. lifting too heavy one-sided movements for too long e.g. driving / washing dishes / vacuuming
5. DK - rengøring - cleaning
6. DK - hvor jeg ikke tænker over hvordan jeg bevæger mig, så får jeg mere ondt, spænder, kan ikke sove, træt, mere ondt- when I don't think about how I move, I get more pain, tense, can't sleep, tired, more pain
7. DK – overbelastning – overloading
8. DK – fysiks aktivitet – physical activity
9. SWE - överansträngning typ böja sig ner för mycket – overexertion – such as bending over too much
10. DK – fysisk belastende arbejde- physically demanding work

11. SWE - överansträngning av led- och muskelfästen - overexertion of joints and tendons
12. Chile – ejercicio – excercise
13. DK- Over aktivitet- Overactivity
14. Chile - depués de actividad física - after physical activity
15. DK- Pludselig krævende fysisk aktivitet feks, havearbejde udløser sygdomsaktivitet. Også at stå med hovedet i længere tid ad gangen- feks hvis jeg luger ukrudt eller vasker/tørrer hår med hovedet nedad.- Sudden demanding physical activity such as gardening triggers disease activity. Also, standing with my head for prolonged periods of time - for example, if I'm weeding or washing/drying my hair upside down.
16. SWE – belastning av leder – strain on joints
17. SWE - överanstängt mig – exhaustion
18. SWE - hög muskel belastning – strain on muscles
19. SWE – jobbar kroppsligt – working with the body
20. SWE – tumme och lillfinger blir stela vid jobb och aktiviteter – thumb and pinky finger get rigid with work and activities
21. SWE – upprepande rörelser - repetitive movements
22. SWE- min artrit förvärras av mycket hårt fysiskt arbete – my arthritis worsens after very hard physical work
23. SWE – racketsporter – racket sports
24. SWE - när jag burit porslin, dukat många tallrikar mm inför samling med 35 personer – when I have carried porcelain, set the table etc before a gathering of 35 people
25. SWE – mycket fysisk aktivitet – alot of physical activity
26. SWE - för mycket aktivitet – too much activity
27. SWE - när jag rört mig för mycket – when I have been too active
28. SWE – Mycket idrott – alot of physical excercise/sports
29. SWE - ökad belastning, för mycket aktivitet – increased strain, too much activity
30. SWE – belastning fysiskt – physical strain
31. SWE – promenader – walks
32. SWE - överbelastning ex i arm – over exertion e.g. in an arm
33. SWE - när jag rört mig mycket, dvs varit ingång större delen av dagen – when I have moved about alot, been active most of the day
34. SWE – tung belastning – heavy strain
35. SWE - överaktivitet - over activity
36. SWE – tung belastning på leder och muskler – heavy strain on joints and muscles
37. SWE – fysisk aktivitet – physical activity
38. SWE – skala potatis – peel potatoes
39. SWE – tagit i för mycket vid träning - when I have put too much effort into training
40. SWE - långa promenader – long walks
41. SWE - hård fysisk belastning – heavy physical strain
42. SWE - träning dvs upprepade rörelser vilket ger mig entesiter – training ie repetitive movements gives me enthesitis
43. SWE – snabba promenader, high tempo walks
44. SWE - långa promenader utan vila – long walks without rest
45. SWE – lederna försämras också av hård belastning – joints worsen with heavy strain
46. SWE - när jag belastar tungt på gymmet – when use heavy weights in the gym
47. SWE - hårt kroppsarbete – tough physical activities
48. SWE – Fysisk aktivitet – physical activity
49. SWE – promenader längre än 200 m – walks longer than 200 m
50. SWE - träning bra men för mycket belastning vid träning triggar igång - excercise is good but too much strain during excercise is a trigger

Q TOBACCO
1. SWE – tidigare rökning - previously smoking
2. Chile – tabaco – tobacco
3. Chile – tabaco – tobacco
4. SWE – tobak rökning passiv – passive smoking

5. SWE – tobak – tobacco
6. NL- roken- smoking
7. Chile – tabaco – tobacco
8. Chile – consumo de tabaco - tobacco use
9. NL – tabak- tobacco
10. DK – tobak- tobacco
11. Chile – tabaco – tobacco
12. DK- rygning- smoking
13. Chile – tabaco – tobacco
14. Chile – tabaco – tobacco15. DK- cigaretter- cigarettes
16. SWE – tobak – tobacco
17. SWE - rökning - smoking
18. DK- (måske tobak)- (maybe tobacco)
19. NL- tabak- tobacco
20. SWE - rökning - smoking
21. SWE – cigaretter – cigarettes
22. Chile – cigarro – cigar/cigarettes
23. NL- roken- smoking
24. Chile – tabaco – tobacco
25. Chile – tabaquismo – tobacco
26. NL- tabak- tobacco
27. Chile – cigarro – cigar/cigarettes
28. NL- roken- smoking
29. SWE - rökning - smoking
30. NL- rookgedrag- smoking behaviour
31. NL- roken- smoking
32. SWE – tobak - tobacco
33. SWE – tobak – tobacco
34. SWE – nikotin – nicotine
35. SWE - tobaksrökning - smoking tobacco
36. SWE – tobak – tobacco
37. SWE – tobak – tobacco
38. SWE - rökning - smoking
39. SWE - rökning - smoking
40. SWE – tobak – tobacco
41. SWE - rökning - smoking
42. SWE – tobak - tobacco
43. SWE - rökning - smoking
44. SWE - rökning - smoking
45. SWE – tobak – tobacco
46. SWE - rökning - smoking
47. SWE – tobak – tobacco
48. SWE - tobak – tobacco
49. SWE - rökning - smoking

R LACK OF EXERCISE

1. Chile – Con la actividad física disminuye. Aumenta con poca actividad.
– With
physical activity it decreases. It increases with little activity.
2. SWE – reducerad fysisk träning - reduced physical activity.
3. Chile – falta de deporte – lack of sports
4. NL- vanwege een achillespeesreptuur de laatste acht maanden niet kunnen sporten. hierna is het extremer geworden- due to an Achilles tendon rupture, unable to play sports for the last eight months. after this, it became more extreme
5. DK- mangel på motion - lack of exercise
6. Chile – falta de ejercicio – lack of exercise
7. NL- te weinig beweging – lack of exercise
8. SWE – stillasittande – being sedentary
9. SWE - väldigt lite rörelse - very little excercise

10. SWE - lägre aktivitetsnivå - lower activity level
11. SWE – om jag inte motionerar – if I do not excercise
12. DK ...hjælper diæt och motion-
...diet and exercise helps.
13. DK- perioder med nedsat motion- periods of reduced exercise
14. DK- Manglende træning/motion- Lack of training/exercise
15. Chile – falta de ejercicio- lack of exercise
16. SWE – missad vattengympa, hoppa över fysisk aktivitet – missed aquarobics, missing physical activities
17. Chile – inactividad, sedentarismo
18. SWE – inaktivitet, när jag inte rört mig på några dagar – physical inactivity, not being acitive a couple of days
19. SWE - låg aktivitetsnivå - low activity levels
20. SWE – inaktivitet – inactivity
21. SWE - nedgång i fysisk form, tränar sämre – lower fitness, worse training
22. SWE - dålig eller ingen träning - bad or lack of training
23. SWE – vid ej motion – no excercise
24. SWE - när jag inte är regelbundet fysisk aktiv – when I am not regularly physically active
25. SWE – brist på träning - lack of excercise
26. SWE – stillasittande – inactivity
27. SWE – stillasittande/stillastående - inacitivity
28. SWE – om jag inte tränar - if I do not excercise
29. SWE – inaktiv – inactivity
30. SWE – Stillasittande – inactivity
31. SWE - för lite rörelse - too little movements
32. SWE – om jag inte tränar - if I do not excercise
33. SWE – mindre träning - less training
34. SWE - när jag inte tränar hårt - when I do not excercise vigorously
35. SWE - för mycket stillasittande – too much inactivity
36. SWE – Om jag inte är fysiskt aktiv – if I am not physically active
37. SWE - för lite rörelse - too little movements
38. SWE – minskad träning - diminished excercise
39. SWE – stillasittande – inactivity
40. SWE – lite motion/träning - little excercise/training
41. SWE – om jag minskar min vardagsträning - if I diminish my daily excercise
42. SWE – brist på motion – lack of excercise
43. SWE - för lite rörelse - to little motion

S WEATHER/CLIMATE
1. DK –kulde – cold
2. Chile - sobrexposición sol – too much sun exposure
3. DK – vejrskift- weather changes
4. NL – weersomstandigheden- weather conditions
5. SWE - väderomslag - sudden weather change
6. DK- vejrskifte- weather change
7. DK- vejrskifte – weather change
8. NL - in de winter de eerste periode dat de verwarming weer aangaat- in the winter the first period when the heating comes back on
9. DK- vejret – weather
10. DK- varme- heat
11. SWE - hög sol, värme - high sun, heat
12. DK- kulde- cold
13. Chile – cuando me encuentro en lugares muy calidos - when I am in very warm places
14. DK- for meget sol- too much sun
15. DK- vejret- weather
16. DK- vejrskifte- change of weather
17. Chile – cambios de temporada – change of season
18. Chile - Frío - cold

19. Chile – clima – climate
20. SWE - förändringar i temperatur och luftfuktighet – changes in temperature and air humidity
21. DK- Pludselige vejrskift- Sudden changes of weather
22. DK- vejrskift- change of weather
23. SWE – kalla månader - cold months
24. NL- weersvandering- weather migration.
25. DK- Estacional- Seasonal
26. Chile - frío - cold
27. Chile – cambios de clima – shifts in climate
28. DK- frostvejr- frosty weather
29. SWE – mindre solljus – less sunlight
30. NL- Winter/kou vochtig weer- Winter/cold humid weather
31. Chile – calor – heat
32. NL- warm (vochtig) weer. Warm (humid) weather.
33. DK- temperaturskift- shift of temperature
34. SWE - dåligt väder - bad weather
35. NL- Vanneer er veel regen valt.- When there is a lot of rain.
36. SWE – kallt väder, minusgrader – cold weather, temperatures below 0
37. DK- perioder uden sol- periods without sun
38. Chile – sol – sun
39. SWE – humidity, temperature
40. Chile – con el calor – with heat
41. SWE – väder - weather
42. Chile – calor frio - heat cold
43. Chile – cambios de temperatura – change of temperature
44. NL- oostenwind- east wind
45. NL- Vochtig weer.- Humid weather.
46. NL- kou- cold47. NL- koud en droogte. Warme en zon is trekt het zo weg en de pijn ook. - cold and dryness. Warmth and sunshine takes it away in no time and also the pain.
48. SWE – kyla – col
49. SWE – kyla och vind – cold and wind
50. DK – Varme, Fugt, Kulde – heat, humidity, cold

T SEASON
1. Chile - época invernal – winter season
2. Chile – invierno – winter
3. NL- in de winter minder zon en droge lucht- less sunshine and dry air in winter
4. Chile – periodo de invierno – winter period
5. DK- vinter- winter
6. SWE – vinter – winter
7. Chile – invierno – winter
8. DK- vinter- winter
9. Chile – estacional – seasonal
10. DK- vinterperioden/koldere vejr- winter period/colder weather
11. DK- vintertiden – winter season
12. SWE - vintersäsong - winter season
13. Chile – en invierno – in winter
14. Chile – primavera – spring
15. Chile – en época de primavera/verano me brota con más fuerza – in spring/summer I have more intense flares
16. NL – in de winter – in winter
17. NL – winterkou – wintercold
18. Chile – con el invierno – in winter
19. SWE – lack of sun (winter)
20. Chile - estación del año - de season of the year
21. DK – vinter – winter
22. NL – winter en weinig zon – winter and lack of sun
23. SWE – vintertid – wintertime
24. SWE – vinter-vår säsong - winter-spring season

25. NL – wisseling van seizoenen – seasonal changes
26. Chile – invierno - winter
27. SWE - säsongs ändringar - seasonal changes
28. NL – vooral in de winter – mainly in winter
29. SWE – vintern – winter
30. SWE - säsongsbyte - seasonal change
31. NL – winter/herfst - winter/autumn
32. Chile – invierno – winter
33. SWE – vinter – winter
34. SWE – jag blir sämre på sommaren – I get worse in summers
35. DK – vinter (mørke) - winter (darkness)
36. SWE – på sommaren, på våren - summers and springtime
37. Chile - estación del año - season of the year
38. SWE – vinter – winter39. SWE- höst o vinter – autumn and winter
40. DK - mørke/vinter - darkness/winter
41. NL – vochtige wintermaanden – moist winter months
42. Chile – el cambio estacional – change of season
43. Chile – estacional – season
44. NL – zomer hitte – heat of summer
45. DK - vinterhalvåret - invernal 6 months
46. Chile - frío (invierno) - cold (winter)
47. NL – Winter – winter
48. SWE – vintertid – wintertime
49. NL – winter – winter
50. NL – winter – winter

U SLEEP DISORDER
1. DK – mangel på søvn- lack of sleep
2. SWE - dålig sömn - bad sleep
3. DK- søvnmangel- lack of sleep
4. DK- dårlig søvn - bad sleep
5. Chile – dormir poco – little sleep
6. Chile – mal dormir – bad sleep
7. DK- søvn problemer – sleep problems
8. NL- weinig slaap- little sleep
9. NL- weinig slaap- little sleep
10. Chile – cuando duermo poco – when I sleep little
11. DK - dårlig nattesøvn - poor sleep at night
12. DK – manglende søvn - lack of sleep
13. DK – manglende søvn - lack of sleep
14. DK – for lidt søvn - too little sleep
15. NL – slecht slapen – poor sleep
16. SWE - sömn - sleep
17. DK – manglende søvn - lack of sleep
18. DK – for lidt søvn - too little sleep
19. SWE – sleep
20. SWE - dåliga sömnvanor - bad sleeping habits
21. SWE – mindre sömn - less sleep
22. SWE - sömnbrist - lack of sleep
23. NL - slaapgebrek – lack of sleep
24. SWE - dålig sömn - bad sleep
25. DK - dårlig søvn - bad sleep
26. DK – nedsat søvn - decreased sleeping
27. SWE - Insömn - insomnia
28. Chile – falta de sueño - lack of sleep
29. SWE - dålig sömn - bad sleep
30. DK – sove for ledt – shallow sleep
31. DK – manglende søvn - lack of sleep
32. SWE – sover dåligt - sleep bad33. DK - søvnmangel - lack of sleep
34. SWE - sömnbrist - lack of sleep

35. SWE - sömnbrist - lack of sleep
36. DK – nedsat søvn - decreased sleeping
37. SWE - sömnbrist - lack of sleep
38. SWE – lite sömn - little sleep
39. NL – te weinig slaap – to little sleep
40. NL – slaap gebrek – lack of sleep
41. SWE - sömn - sleep
42. SWE - sömnproblem - sleeping problems
43. SWE - sömnbrist - lack of sleep
44. SWE - dålig sömn - bad sleep
45. SWE - dålig nattsömn - bad nocturnal sleep
46. SWE - dålig sömn - bad sleep
47. SWE – insomnia
48. SWE- sömnbrist - lack of sleep
49. SWE – lite sömn - little sleep
50. SWE - sömnbrist - lack of sleep

V KOEBNER
1. NL- zonnebrand- sunburn
2. DK- tattoos- tattoos
3. NL- duidelijke contactgevoelig. plekken waar een horloge voortdurent beweegt of een ring. of bij irritatie in de oksels, dit is inmiddels opgelost door gebruik van zoutsticks als deodorant- clearly contact-sensitive. places where a watch constantly moves or a ring. or for irritation in the armpits, this has since been solved by using salt sticks as deodorant
4. NL- wondje- wound
5. SWE – solsveda – sunburn
6. SWE- kläder som river upp placken- clothes that rip up the plaques
7. SWE- sårskada- wound injury
8. Chile – quemaduras solares – sunburn
9. NL- wondjes- wounds
10. NL - zonnebrand- sunburn
11. SWE – solsveda, tatueringar – sunburn, tattoos
12. Chile – quemaduras solares – sunburn
13. NL – zonnebrand – sunburn
14. DK – nye sår/rifter - new wounds/tears
15. DK - forbrænding - burns
16. SWE - solbränna - sunburn
17. NL – zonnebrand – sunburn
18. SWE – riva/klia/gnugga - tearing/scratching/rubbing
19. SWE - långvarig påfrestning av hudområde - long strain on a skin area
20. DK – solskoldning – sunburn
21. Chile – heridas – wounds22. NL – teen nagel gestoten daardoor psoriasis teen nagel – toe nail trauma led to nail psoriasis
23. SWE – skav – rubbing
24. SWE – massor av myggbett – multiple mosquito bites
25. SWE – om jag bränner mig i solen, skär mig vid mattlagning, river huden i buskarna vid rabatt ränsning - when I burn myself in the sun, cut myself when cooking, scratch myself on bushes whilst weeding in the garden
26. DK – solskoldning – sunburn
27. SWE – solsveda – sunburn
28. NL – tattoo – tattoo
29. NL – insektensteek – insect bites
30. NL – zonnebrand – sunburn
31. NL – injecteren voor diabetes – injections for diabetes
32. SWE – river för hårt när det kliar – scratch too hard when it itches
33. NL – Een te harde riem die te strak zak, een stoel met scherpe armleuningen. Op knieën als ik kniel met dweilen en stofzuigen onder het bed – a belt that was too hard and too tight, a chair with sharp armrests. On my knees when I kneel during cleaning or vacuuming under the bed.

34. SWE – Det blir psoriasis av att jag kliar mig – psoriasis appears where I scratch myself
35. SWE – sko som skaver - chafed feet
36. SWE – nötning vid troskanter, eller annan påfrestning på hudpartier, när jag slagit i huvudet i skåplucka, rispat mig, ibland tom vid myggbett - strain on skin from waistbands, other strains on skin areas, when I have hit my head on cupboard door, scratched myself, sometimes even with mosquito bites
37. SWE – skaver – scratches
38. SWE - nötning - wear
39. SWE – solsveda - sunburn
40. SWE – tatuerat mig – got a tattoo
41. SWE – hantering av grova eller grovt fibrösa ytor (t.ex. Salta tampar på båten) - handling of rough and fibrous surfaces (e.g. salty ropes on the boat)
42. SWE - nötning - wear
43. SWE - för mycket sol – too much sun
44. SWE – solsveda – sunburn
45. SWE – skav, nötning, skada – scratch, wear, injury
46. SWE - När jag var 14 år ramlade jag med cykeln och slog mig i huvudet. Efter det fick jag psoriasis i hårbotten - When I was 14 years old I fell with my bicycle and hit my head. After that I got scalp psoriasis.
47. SWE – arbete med händerna kan ge problem på händerna - working with the hands can give problems on the hands
48. SWE – skada – injury
49. SWE – stukning av fotled – spraining of ancle
50. SWE – att jag kliar – when I scratch

W SKIN CONTACT
1. DK- cremer mod psoriasis- creams against psoriasis2. NL- geparfumeerde créme- perfumed cream
3. 57NL- chloor- chlorine
4. Chile - reacción a algún material – reaction to some material
5. NL- sommige zonnebrandcreme- some sunscreen
6. DK- såpe med parfyme- soap with perfume
7. NL- soms reageer ik wel slecht op bepaalde gezichtsmiddelen zonder verklaring. ik bin niet allergisch voor iets - sometimes I do react badly to certain face products without explanation. i am not allergic to anything.
8. Chile – agua de mar – sea water
9. SWE – vissa kemikalier i krämer - certain chemicals in lotions
10. DK - parfume i creme og shampoo- perfume in cream and shampoo
11. NL – na het gebruiken van producten zoals shampo/douchegel/bodylotion - after using products such as schampoo/showergel/bodylotions
12. NL – bepaalde wasmidel – certain detergents
13. NL – strakke kleding (schurende kleding), polyester kledij - tight clothing, polyester clothing
14. SWE - sköljmedel - softeners
15. DK – skift af deodorant – change of deodorant
16. NL – haarverf en gebruik van zelfbruiner – hair dye and use of self-tanner
17. NL – wasmiddel met parfum, docheprodukten met parfum – detergent with perfume, perfumated shower products
18. NL – douche gels – shower gels
19. SWE – kontakt med vatten – water contact
20. SWE – simning i bassäng - swimming in pool
21. SWE – strumpor, varm och fuktig om fötterna, om jag är i vatten med händerna - socks, warm and moist feet, if I have my hands in water
22. SWE - när jag utsätter fötterna för vatten – when I expose my feet to water
23. SWE - tvättning av händer - washing hands
24. SWE – handsprit – hand desinfectants
25. SWE – kallt vatten när man sköljer saker eller badar – cold water when rinsing things or bathing
26. SWE - ullkläder, åtsittande kläder - wool clothing, tight clothing
27. SWE – vissa hårprodukter - some hair products

28. SWE - täta handskar – tight gloves
29. SWE – hygienprodukter – hygienproducts

X UNCATEGORISED

1. Chile – falta de consumo de agua – lack of water consumption
2. NL- materialen van kleding, bedekken van de huid/afsluiten- material of clothing, covering the skin/sealing
3. NL – harddrugs- hard drugs
4. SWE – slutade röka - stopped smoking
5. SWE – osund livstil – unhealthy lifestyle
6. Chile – sistema nervioso alterado – altered nervous system
7. Chile – menos consumo de agua – less water consumption
8. DK- da jeg stoppede med at ryge brød det volsomt ut- when I quit smoking, it broke out violently
9. Chile – sudor – sweat
10. Chile – estando sin hacer nada – doing nothing
11. SWE - viktnedgång - loosing weight
12. SWE – livet – life
13. Chile - después del baño - after bathing
14. NL – gewichtverlies ook – weight loss
15. SWE – ingen tillgång till bad – no access to baths
16. SWE – pollen – pollen
17. Chile – poco descanso – lack of rest
18. SWE – varma eller syntetiska klädmaterial - varm or synthetic clothing materials
19. DK – ingen vinterbadning – no winter bathing
20. DK - strømper - socks
21. NL – tandpasta? - toothpaste?
22. NL – verandering van het bloed om de 7 jaar – change of the blood every 7 years
23. SWE – svettningar – sweating
24. SWE – pollen säsongen - pollen season
25. SWE – när jag slutade röka 1993 blossade psoriasen up – when I stopped smoking 1993 my psoriasis flared
26. SWE – varma kläder/sängkläder - warm clothes or bedding
27. SWE – behandling – treatment
28. SWE - rökarom - smoke aroma
29. SWE - pollensäsongen - pollen season
30. SWE - hårt vatten – hard water
31. SWE – varmt vatten, varma kläder - warm water, warm clothing
32. SWE - när jag tar på saker – when I touch things
33. SWE – svettning – sweating
34. SWE – strumpor, varm och fuktig om fötterna - socks, warm and moist feet
35. SWE – utmattning – exhaustion
36. SWE - förändringar i levnadsvanor – changes in lifestyle
37. SWE - försenad blodgivning – delayed blood donation
38. SWE - när jag är utmattad – when I am exhausted
39. SWE - när man svettas – when one sweats
40. SWE – resor – travel
41. SWE – dehydrering – dehydration
42. SWE - vätskebrist - dehydration
43. SWE – torr hud – xerosis
44. SWE – vatten – water
45. SWE - lågtryck - low pressure
46. SWE – att inte duscha/tvätta håret - not showering/washing hair
47. SWE – klor – chlorine
48. SWE - långvarig plåster efter operation – long bandaging after surgery
49. SWE - hög aktivitetsnivå - high activity level
50. SWE – klor – chlorine

Y MISSING DATA
