# Supplementary material for: Psoriasis Triggers and Disease Activity: Analysis of Survey Data from the PSODEEP1 Study
Source: Acta Derm Venereol. 2026 Jun 1;106:0167. doi: 10.2340/actadv.v106.adv-2025-0167 (PMC13224730; doi:10.2340/actadv.v106.adv-2025-0167)
Supplement: Supplementary Material 1. [file ActaDv-106-0167-s0002.pdf]

Supplementary material has been published as submitted. It has not been copyedited, or typeset by Acta Dermato-Venereologica

Fig. S1. Stacked bar chart showing temporal occurrence of disease flare-up or disease relapse (periods of worsening symptoms) over the last 3 years. * number of participants to this specific questionnaire item.

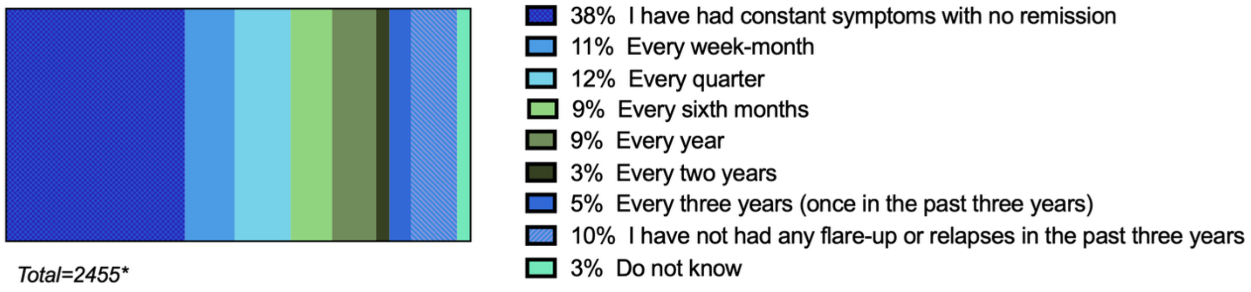

Fig. S2. Bar chart showing prevalence of self-reported seasonal variation in psoriasis disease activity by country. "Do you experience seasonal variation in your psoriasis (e.g. improvement in summer and worsening during winter?"

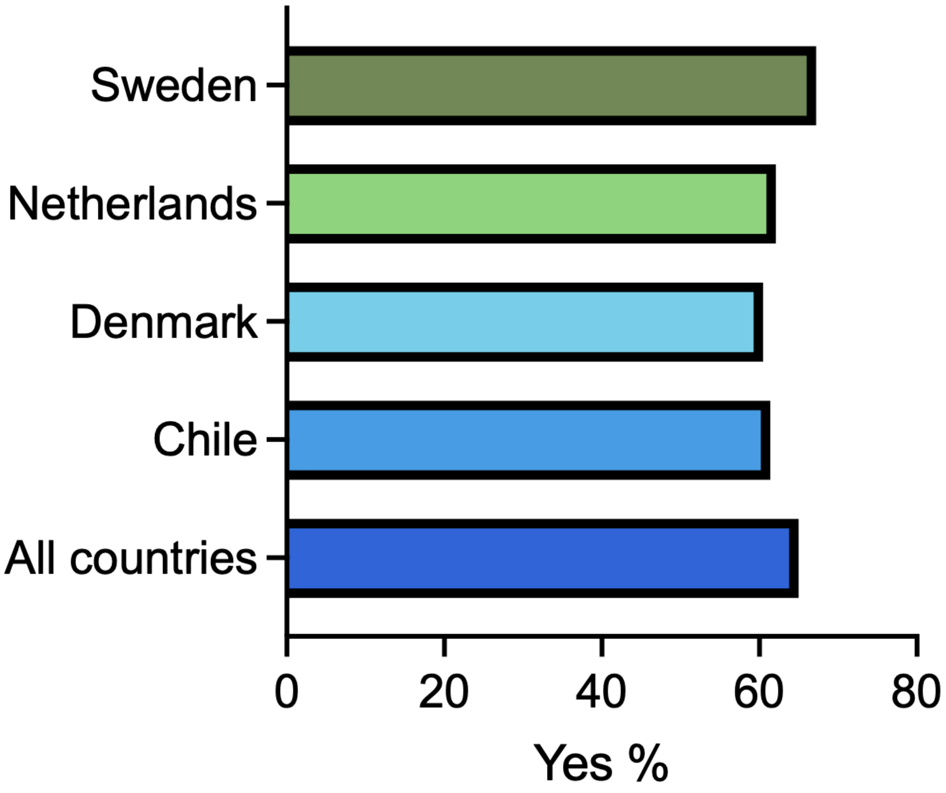

Fig. S3. Stacked bar chart showing the number of reported triggers by study participants.

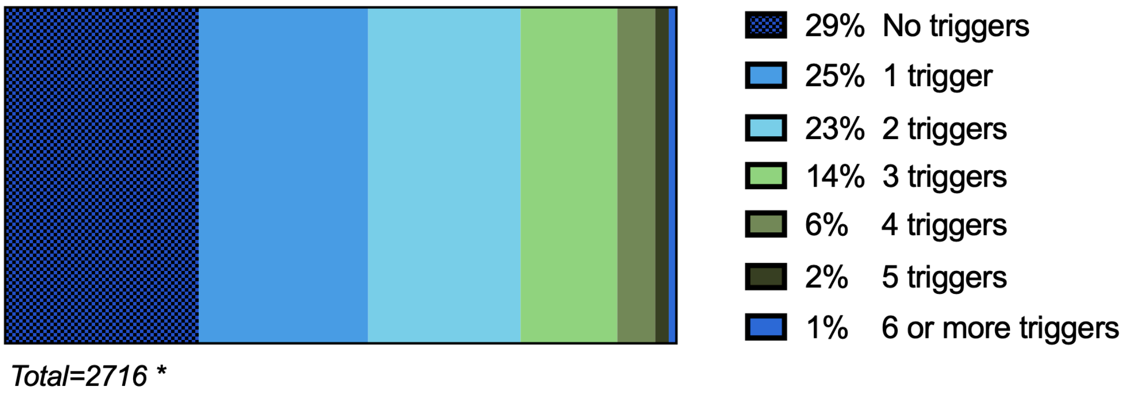

Fig. S3 Stacked bar chart showing the number of reported triggers by individuals. * 41 respondents acknowledged having disease triggers but failed to specify these being included in "No triggers".

Fig. S4. Top 10 Self-reported triggers subgroups divided by individuals having psoriatic arthritis.

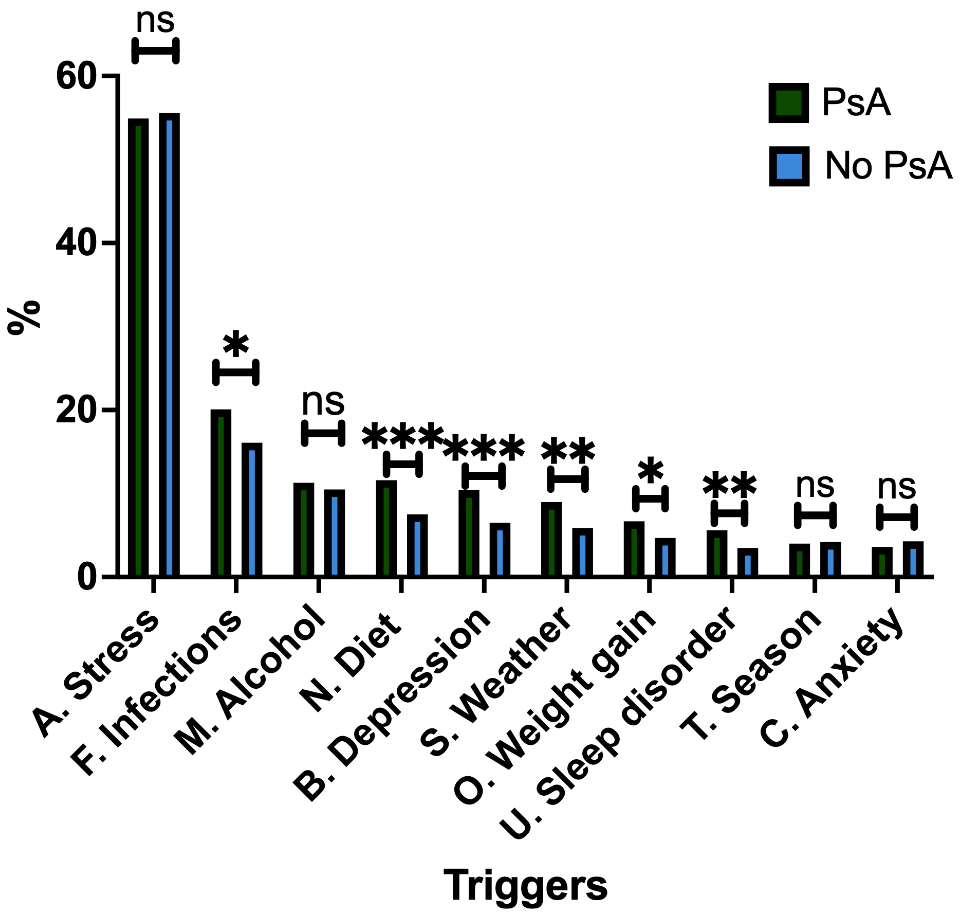

Figure S4. Top 10 Self-reported trigger subgroups divided by individuals having psoriatic arthritis (PsA). *Chi*-squared (χ^2) test, * p<0.05, ** p<0.01,*** p<0.001
